# Emerging Role of Purine Metabolizing Enzymes in Brain Function and Tumors

**DOI:** 10.3390/ijms19113598

**Published:** 2018-11-14

**Authors:** Mercedes Garcia-Gil, Marcella Camici, Simone Allegrini, Rossana Pesi, Edoardo Petrotto, Maria Grazia Tozzi

**Affiliations:** 1 Dipartimento di Biologia, Unità di Fisiologia Generale, Via San Zeno 31, 56127 Pisa, Italy; mercedes.garcia@unipi.it; 2 Dipartimento di Biologia, Unità di Biochimica, Via San Zeno 51, 56127 Pisa, Italy; simone.allegrini@unipi.it (S.A.); rossana.pesi@unipi.it (R.P.); edoardo.petrotto@gmail.com (E.P.); maria.grazia.tozzi@unipi.it (M.G.T.)

**Keywords:** cytosolic 5′-nucleotidase II, adenosine kinase, adenosine deaminase, hypoxanthine guanine phosphoribosyl transferase, xanthine oxidase, uric acid

## Abstract

The growing evidence of the involvement of purine compounds in signaling, of nucleotide imbalance in tumorigenesis, the discovery of purinosome and its regulation, cast new light on purine metabolism, indicating that well known biochemical pathways may still surprise. Adenosine deaminase is important not only to preserve functionality of immune system but also to ensure a correct development and function of central nervous system, probably because its activity regulates the extracellular concentration of adenosine and therefore its function in brain. A lot of work has been done on extracellular 5′-nucleotidase and its involvement in the purinergic signaling, but also intracellular nucleotidases, which regulate the purine nucleotide homeostasis, play unexpected roles, not only in tumorigenesis but also in brain function. Hypoxanthine guanine phosphoribosyl transferase (HPRT) appears to have a role in the purinosome formation and, therefore, in the regulation of purine synthesis rate during cell cycle with implications in brain development and tumors. The final product of purine catabolism, uric acid, also plays a recently highlighted novel role. In this review, we discuss the molecular mechanisms underlying the pathological manifestations of purine dysmetabolisms, focusing on the newly described/hypothesized roles of cytosolic 5′-nucleotidase II, adenosine kinase, adenosine deaminase, HPRT, and xanthine oxidase.

## 1. Introduction

**Figure 1:**
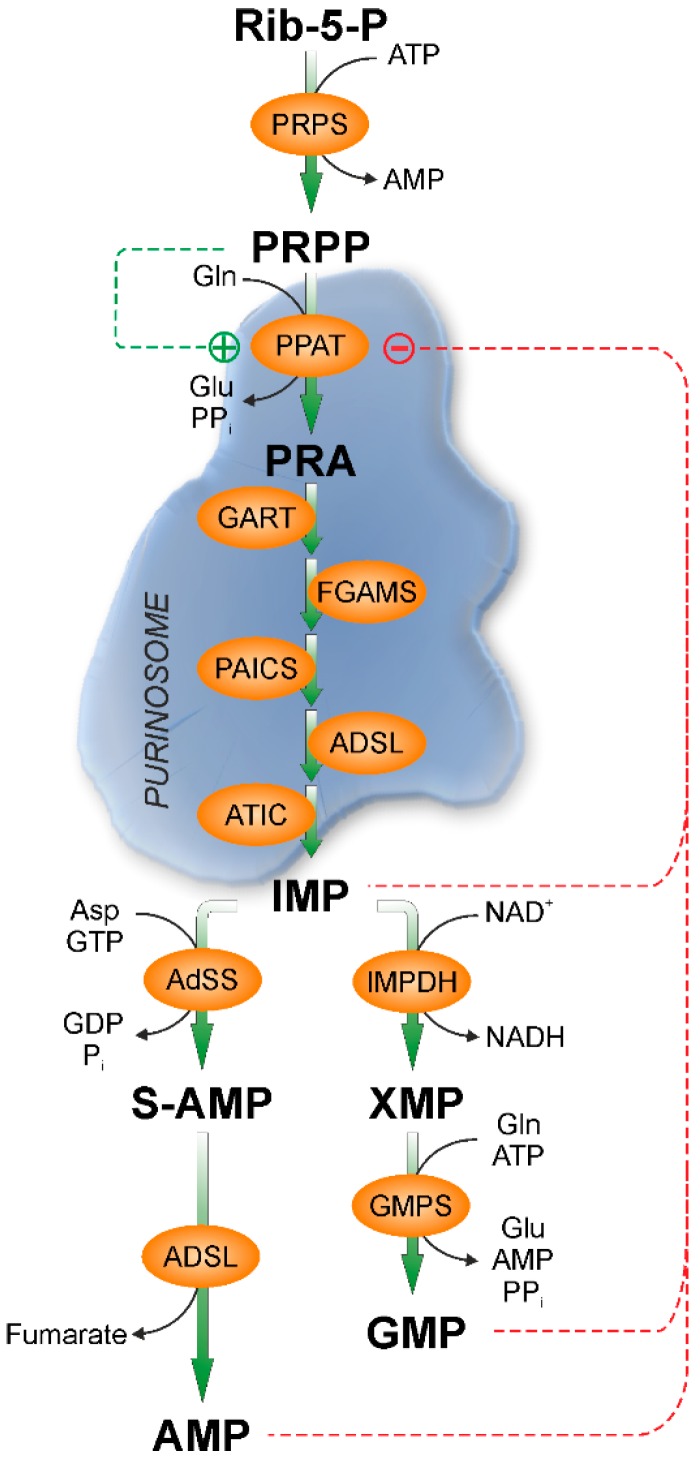
Purine de novo synthesis and its regulation. PRPP (5-phosphoribosyl-1-pyrophosphate) is synthesized from ribose-5-phosphate (Rib-5-P) by PRPS (PRPP synthetase). Six enzymes catalyze the ten steps required to convert PRPP into IMP: PPAT (PRPP amidotransferase), trifunctional GART (phosphoribosylglycinamide synthetase/phosphoribosyl glycinamide transformylase/ phophoribosyl aminoimidazole synthetase), FGAMS (phosphoribosyl glycinamidine synthase), bifunctional PAICS (phosphoribosyl aminoimidazole carboxylase/phosphoribosyl aminoimidazole succinocarboxamide synthetase), ADSL (adenylosuccinate lyase), and bifunctional ATIC (5-aminoimidazole-4-carboxamide ribonucleotide transformylase/IMP cyclohydrolase). IMP is converted to IMP and GMP. AdSS: Adenylosuccinate synthase, IMPDH: IMP dehydrogenase, GMPS: GMP synthetase. PRA: phosphoribosylamine. +: activation; −: inhibition.

**Figure 2:**
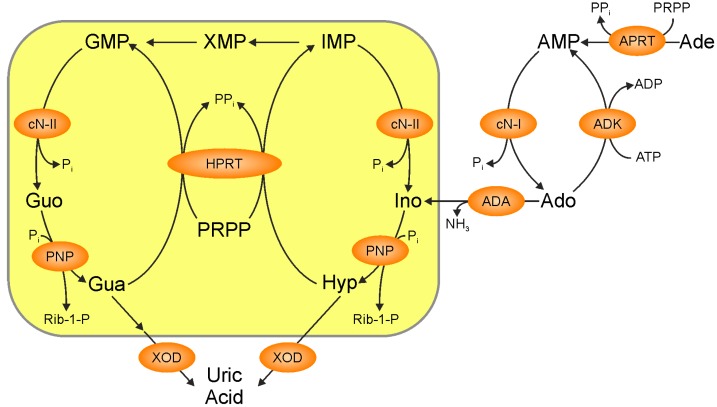
Purine salvage and catabolism. APRT: adenine phoshoribosyltransferase, ADK: adenosine kinase, ADA: adenosine deaminase, cN-I: cytosolic 5′-nucleotidase I, cN-II: cytosolic 5′-nucleotidase II, HPRT: hypoxanthine-guanine phosphoribosyltransferase, PNP: purine nucleoside phosphorylase, XOD: xanthine oxidase, Ade: adenine, Ado: adenosine, Gua: guanine, Guo: guanosine, Hyp: hypoxanthine, Ino: Inosine, PRPP: 5-phosphoribosyl-1-pyrophosphate, Rib-1-P: ribose-1-phosphate. The yellow box: the purine cycle.

Purine and pyrimidine nucleotides, nucleosides, and bases play several very important roles in cells. It is universally accepted that their synthesis from amino acids and sugars occurs mainly during the G1 and S phases of cell cycle to meet the high nucleotide demand during proliferation [1]. Nonproliferating cells, which need a smaller amount of nucleotides, rely mainly on catabolism and salvage, and in complex organisms these two pathways not always take place in the same cell or organ [2]. Enzymes involved in both de novo and salvage pathways are synthesized in greater amount during G1/S and early S phases of cell cycle [3]. The flux of substrates and intermediates through the de novo pathway is regulated mainly by the aggregation of a purinosome that, bringing together the enzymes involved in the pathway, strongly increases the rate of purine synthesis. In fact, it was demonstrated that the six enzymes catalyzing the 10 steps of purine biosynthesis, can cluster near mitochondria and microtubules to form dynamic multienzyme complexes referred to as a “purinosome” [4]. The purinosome aggregation is driven by the absence of preformed purines in the medium, indicating that both de novo and salvage pathways cooperate to meet the objective to supply the correct amount of nucleotides, nucleosides, and bases for the cell requirements in any phase of the cell cycle [1]. The purinosome assembly is also dependent on several distinct proteins such as Hsp90, casein kinase II, and hypoxanthine guanine phosphoribosyltransferase (HPRT) and also on small compounds such as inhibitors of microtubule polymerisation and G-protein coupled receptors agonist, as well as on mitochondrial performances [1]. De novo synthesis is feedback regulated by its final products adenosine 5′-monophosphate (AMP), inosine 5′-monophosphate (IMP) and guanosine 5′-monophosphate (GMP), which are allosteric inhibitors of 5-phosphoribosyl-1-pyrophosphate (PRPP) amidotransferase (PPAT) [5], while it is increased in the presence of PRPP which is both substrate and allosteric activator of the same enzyme [6] (Figure 1). Conversely, the purine salvage pathway is mainly regulated by the level of expression of enzymes and their kinetic characteristics and substrate concentrations, particularly of PRPP, which is a cosubstrate for adenine, hypoxanthine, and guanine salvage [7] (Figure 2). Therefore, PRPP appears to be an important regulator in common to both pathways [6,7]. As shown in Figure 2, the purine salvage pathway and purine catabolism are strictly correlated. The first catabolic step is catalyzed by 5′-nucleotidases which generate nucleosides from nucleoside monophosphates. Among the six cytosolic isoenzymes of 5′-nucleotidases, cytosolic 5′-nucleotidase-I (cN-I) (specific for AMP) and cytosolic 5′-nucleotidase II (cN-II) (specific for IMP-GMP) are allosterically regulated by adenylic compounds [8,9]. While cN-I is mainly expressed in muscle, cN-II is an ubiquitous, highly regulated, and structurally conserved enzyme present in all the eukaryotic organisms with the exception of yeasts [8]. It has been demonstrated that cN-II activity is involved in the regulation of concentration of purine compounds, ribose-1-phosphate, and PRPP as a function of energy charge and of phosphorylated compounds such as 2,3 bisphosphoglycerate and diadenosine tetraphosphate [8]. In fact, cN-II is the regulated enzyme in the IMP-GMP cycle (Figure 2). In the presence of allosteric activators, cN-II dephosphorylates IMP or GMP, generating inosine or guanosine, which in turn can be split into ribose-1-phosphate and hypoxanthine or guanine. The purine bases are in part salvaged back to IMP or GMP and in part discharged as such or as uric acid, while ribose-1-phosphate can be utilized to salvage purine or pyrimidine bases, through the action of nucleoside phosphorylases, or isomerized into ribose-5-phosphate and then utilized for PRPP synthesis [10]. CN-II is usually highly expressed in cells and organs with high nucleic acid turnover and in tumors [8]. The effect of cN-II silencing or hyperexpression has been studied in several tumor cell models [11,12,13], demonstrating that the enzyme hyperexpression is followed by a strong decrease of all triphosphorylated purine and pyrimidine nucleosides [14]. Conversely, cN-II silencing causes an increase of the intracellular concentration of the same compounds [11,14]. In some cell models, the level of cN-II expression reflects on proliferation and on drug resistance [15]. All of these observations indicate that cN-II plays a fundamental role in the regulation of nucleotide concentration inside the cell probably through both its hydrolytic activity and the regulation of intracellular PRPP concentration. Among the purine nucleosides generated by hydrolysis of nucleoside monophosphate catalyzed by cytosolic 5′-nucleotidases, adenosine is the only one that can be phosphorylated back to nucleoside monophosphate, by adenosine kinase (ADK) located in the cytosol [16]. Deoxyguanosine kinase activity is present and active in mitochondria [17], but no kinase activities specific for guanosine or inosine are present in the cytosolic compartment of eukaryotic cells [18]. Adenosine can be generated outside or inside the cell by nucleotide dephosphorylation, can cross the membrane following its concentration gradient and plays a number of regulatory roles through the interaction with specific receptors (A1R, A2AR, A2BR, and A3R). Typically A1R and A3R are coupled with the G_i/o_ family of G-proteins which inhibit cyclic AMP production, whereas A2AR and A2BR stimulate cyclic AMP production via G_s_. Furthermore, all the adenosine receptors activate at least one subfamily of mitogen activated protein kinases (MAPK) [19]. Accordingly, adenosine exerts a multitude of functions such as modulation of neuron–glia signaling and neurodevelopment [20]. Furthermore, the nucleoside plays an important role in the control of innate and adaptive immune system [21]. As mentioned above, extracellular adenosine can arise from the catabolism of extracellular ATP. ATP is secreted by a number of cell types and can interact with two families of specific receptors: P2XR, ion-gated channels with neuromodulatory functions, and P2YR, coupled with G-proteins. Purinergic signaling is involved in neuron–glial interactions [22] as well as in learning, memory, sleep, locomotor activity, mood, motivation, and in pathological brain function such as trauma, ischemia, stroke, neurodegenerative diseases, and neuropsychiatric disorders [23,24,25,26,27,28]. Adenosine, inside the cell can be directly phosphorylated by ADK or deaminated by adenosine deaminase (ADA) into inosine, that can in turn be converted into hypoxanthine and then salvaged as IMP or leave the cell as hypoxanthine or uric acid (Figure 2) [29]. The final product of purine catabolism is uric acid that, far from being a mere catabolite to be excreted, has known alternate fame and fortune. It has been described as a very good antioxidant, able to substitute for ascorbate that cannot be synthesized in humans, for the protection of different cells and organs from reactive oxyradicals [30]. Conversely, many epidemiological studies have shown the relationship between uric acid and different disorders such as obesity, metabolic syndrome, hypertension, and coronary artery disease [31]. Clinicians and investigators recognized serum uric acid concentration as a very important diagnostic and prognostic factor of many multifactorial disorders [32]. In this review, we intend to describe the more recent results on the impact of cN-II, ADA, ADK, HPRT, and xanthine oxidase (XOD) activities and their relationship with the molecular mechanisms leading to several pathologies.

## 2. Cytosolic 5′-Nucleotidase II (cN-II)

### 2.1. CN-II and Spastic Paraplegia

As already mentioned, cN-II is an essential enzyme for the regulation of purine compound pool. Recent findings indicate a possible alternative way of action of the enzyme. In fact, an interaction between cN-II and ice protease-activating factor (Ipaf) [33] has been established, which allows us to hypothesize that cN-II, via interaction with Ipaf, may be involved, at some level, in the regulation of the inflammatory process. 

To date, the direct involvement of cN-II into a clearly defined pathology has been assessed in just one single case: autosomic recessive spastic paraplegia 45 (SP45) [34,35,36,37,38]. Hallmark features of all hereditary spastic paraplegias (HSPs) are axonal degeneration and progressive lower limb spasticity caused by loss of corticospinal tract function and significant corpus callosum and white matter pathology. Based on their additional manifestations, HSPs are further classified as uncomplicated or complicated. Complicated HSPs often exhibit different grades of intellectual disability, epilepsy, optic atrophy, deafness, peripheral neuropathy, ataxia, and skin abnormalities [37]. Dursun et al. [34] first reported five patients suffering from SP45 all born from consanguineous unaffected parents. The authors did not relate the pathology directly to *NT5C2* (the gene coding for cN-II), but they mapped the genes responsible for SP45 in the area 10q24.3-q25.1. A few years later, Novarino et al. [35] identified in the same family a truncating mutation at the very N-terminal of cN-II (R29→Stop). In the same study the authors described four other families in which different mutations in *NT5C2* gene (one frameshift-mutant, two splice-mutants, and another stop-mutant) were responsible for SP45 phenotype in homozygous subjects whereas the heterozygous parents did not show any of the features. Since then, three more reports have associated cN-II mutations with SP45 [36,37,38]. The first one described a Qatari family with a mutation in intron 14 leading to an alternative splicing of mRNA with the production of a shorter form of cN-II missing the 58 amino acids of exon 14 (G330 to S387) without any downstream frameshift [36]. Nevertheless, the expected shorter form of cN-II was not detectable in immunoblots of blood samples of affected individuals. Furthermore, the heterologous expression of the mutated cN-II revealed high instability of this enzyme form. Three more individuals of Arab origin suffering from SP45 (all of them offspring of consanguineous marriages) have been described by Straussberg et al. [37]. The exome sequencing performed on nine individuals of this family revealed the expected pattern of a recessive inheritance of a new variant of cN-II, the first missense mutation described, in which a leucine is substituted by a proline (c.1379C > T; p.Leu460Pro). The last reported case of association of cN-II with SP45 described a new cN-II variant in which a 1954 bp deletion in the *NT5C2* gene led to the loss of exon 11 with the consequent deletion of 14 amino acids in cN-II enzyme sequence (p.Lys258_Lys271del). This cN-II variant was found in three siblings of an Iranian family, who exhibited the classic symptoms of complicated SP45 [38]. It would have been very interesting to measure the residual cN-II activity in the patients suffering from SP45. Unfortunately, these data are not available, it is therefore impossible to assess the impact of cN-II mutations on the regulation of intracellular purine and pyrimidine compounds (see Figure 2) and the link between the fluctuations in nucleotide concentration and SP45. In this regard, it is worth mentioning that in another central nervous system (CNS) pathology, Lesch–Nyhan disease (LND) (see below in this review), a significant hyperexpression of cN-II in red cells and fibroblasts was reported [39].

### 2.2. CN-II and Cancer

Many papers demonstrate that cN-II activity is involved in resistance to a number of nucleoside analogs utilized as drugs in the therapy of several kind of tumors, mainly hematological malignancies such as acute myeloid leukemia (AML), chronic lymphoid leukemia (CLL) or acute lymphoblastic leukemia (ALL), but also of some solid tumors, such as non-small cell lung cancer [40,41]. The resistance occurs since cN-II activity can dephosphorylate the phosphorylated form of the nucleoside analog which represents the active drug [15]. Many studies have been performed on patients carrying either alteration in expression levels or mutant forms of cN-II, and on the clinical relevance that these mutants could have on the outcome of the treatment with several nucleoside analogs (mainly cladribine, fludarabine, cytarabine, gemcitabine, 6-mercaptopurine (6-MP), or 6-thioguanine). For a better insight on this matter the reader is referred to exhaustive previously published reviews [15,40,42]. Herein we discuss the latest reports on the subject that greatly contribute to elucidate the role of cN-II into the relapses of ALL treated with thiopurines. 

Tzoneva et al. [43], using a knock-in mouse model for expression of mutant cN-II-(R367Q), the most frequent of the 32 different cN-II mutant alleles to date described in relapse-ALL, demonstrated that this gain-of-function mutant induced resistance to 6-MP both in vitro and in vivo. Leukemia cells expressing cN-II-(R367Q) treated in vitro with 6-MP showed an evident resistance to the purine analog when compared to the cells expressing wild type cN-II (control cells). Moreover, the treatment with 6-MP of leukemic mice harboring the mutant allele of cN-II failed to cause remission, while control mice had a clear dose-dependent response to the drug. These results led the authors to hypothesize that these gain-of-function mutant alleles were responsible in vivo for the resistance to chemotherapy with 6-MP in patients harboring these mutations, being also the cause of the early relapses. Indeed, using the droplet PCR analysis, they also showed in ALL lymphoblasts the presence of two more mutated gain-of-function alleles, P414A and R39Q, during complete remission period, 37 days before the onset of relapse. Mutant R39Q was detectable also in samples taken at the date of the diagnosis, even though in very low amounts. The authors pointed out that, in the absence of 6-MP, T-ALL cells harboring the R367Q mutation proliferated in vitro considerably slower when compared with control cells and in vivo had both delayed entry in S phase and tumor progression. Moreover, lymphoblasts carrying R367Q exhibited a 17-fold reduction, compared to control cells, in the capability to initiate leukemia in transplantation experiments. The authors explain this loss-of-fitness of the R367Q phenotype with the depletion of the cN-II substrates (IMP, XMP, and GMP) with a consequent increased release into the media of the final products of the catabolism (inosine, guanine, hypoxanthine, xanthosine, xanthine, and uric acid). According to the authors, this cN-II gain-of-function mutant made the T-ALL lymphoblasts much more dependent on the rate of purine de novo synthesis, and therefore much more sensitive to drugs targeting this pathway. A series of experiments, both in vitro and in vivo, with several gain-of-function mutants (R367Q, R238W, K359Q, and D407A) clearly demonstrated that cells harboring the mutations were much more sensitive than control cells to the action of mizoribine, an inhibitor of IMPDH, the enzyme responsible for the replenishment of the guanylate pool (Figure 1). Moreover, supplementation of guanosine in the culture media suppressed the effect of mizoribine. In vivo, treatment with mizoribine induced an evident anti-leukemic response in mice with R367Q phenotype when compared with control mice, thus confirming their hypothesis [43]. 

Very recently, the structure of cN-II occurring in relapse leukemic cells was studied in order to define the structural bases of the mutant gain-of-function enzymes [44,45]. In the absence of ATP, the specific activity of cN-II is very low. In fact, the binding of the nucleotide drives a conformational change toward a more active enzyme form [8]. The gain-of-function mutations of cN-II are all involved in the stabilization of the structure of the G355-Q364 region (helix A) in an ordered α-helix form. This region of the protein in the apo-form of control enzyme (not bound to ATP) has a loop structure which turns to an α-helix form upon binding of ATP. This change of conformation facilitates the binding of the substrates and the following catalysis [46]. Considering the heterozygosity of the mutations in ALL, Hnizda et al. [44] and Dieck et al. [45] evaluated the activity of heterooligomeric complexes formed by control and mutated subunits. Their in vitro and cellular studies demonstrated that when a heterooligomer is formed, the activation state is transmitted from the mutated to the control subunit [44]. 

Owing to the high cN-II expression observed in tumor cells and its involvement in drug resistance, cN-II inhibition has been proposed as new therapeutic approach in tumors [15]. To support this idea, cN-II silencing or hyperexpression has been performed in several cancer cell models [11,14]. In the ADF glioblastoma cell line, cN-II hyperactivity was accompanied by an increase of proliferation and resistance to gemcitabine and mitomycin C, while enzyme silencing caused a decrease of cell proliferation [13]. Also in A548 lung cancer cell line, cN-II silencing caused a decrease of proliferation and a metabolic switch from a more glycolytic to a more oxidative phenotype [11]. Finally, in a breast cancer cell model (MDA-MB-231), cN-II silencing caused the activation of a complex mechanism leading to an increase of antioxidant defenses and to a better capacity to respond to a low glucose environment [12]. These very recent results indicate that cN-II is, directly or indirectly, related to regulatory mechanisms that still need to be elucidated. 

### 2.3. CN-II and Other Pathologies

The presence of single nucleotide polymorphisms (SNPs) in the *NT5C2* gene (most of them in untranslated regions) has been associated with many different pathologies, such as schizophrenia, cardiovascular diseases, and others. Nevertheless, most of these reports are obtained from genome-wide association studies (GWAS) and are not supported by more detailed studies. For more information on this subject the reader is referred to the following databases: MalaCards (https://www.malacards.org/), GWAS (https://www.ebi.ac.uk/gwas/), and Open Target Platform (https://www.targetvalidation.org/). 

## 3. Adenosine Kinase (ADK) and Adenosine Deaminase (ADA)

### 3.1. ADK

ADK is an abundant enzyme in mammalian tissues that catalyzes the transfer of the gamma-phosphate from ATP to adenosine, thereby acting as a potentially important regulator of concentrations of both extracellular adenosine and intracellular adenine nucleotides. ADK expression changes during brain development—being neuronal at the first stages and enriched in astrocytes later [47,48]—and in response to brain injury [49,50]. Human ADK consists of two alternatively spliced forms which differ only at the 5′-end. Both isoforms show identical kinetics and both require Mg^2+^ for activity [51]. ADK-large is nuclear and ADK-short is cytoplasmic. Kiese et al. [50] found developmental downregulation of nuclear *Adk*-Large transcripts in neurons and upregulation of cytoplasmic *Adk*-Short transcripts in astrocytes. 

### 3.2. ADK and Neurological Diseases

ADK mutations or alterations in ADK expression have been involved in several pathologies including neurodevelopmental delay [52,53,54,55], epilepsy (reviewed in [56]) and gliomas [57,58]. Recently, upregulation of ADK has been found in reactive astrocytes and a subpopulation of neurons in lesions of Rasmussen encephalitis, a rare neurological disorder characterized by unihemispheric inflammation, progressive neurological deficits, and intractable focal epilepsy [59,60]. Similarly, upregulation of ADK has been reported in surgically resected human epileptic cortical specimens from focal cortical dysplasia type IIB with balloon cells [61]. This dysplasia is a developmental malformation of the cerebral cortex that is associated with pharmacoresistant epilepsy [62]. It has been proposed that dysfunction of adenosine signaling is common in neurological conditions and that it can explain comorbid phenotypes [63] such as epilepsy, Parkinson’s disease and Alzheimer’s disease (AD) among others.

Transgenic mice with ADK knockout or hyperexpressing ADK through all the brain or in specific regions have been a useful tool to uncover the role of ADK in synaptic plasticity, learning, and epilepsy [64,65,66]. Transgenic mice with brain-wide or telencephalon ADK hyperexpression showed dysregulation of brain adenosine which results in working memory deficiency and impaired Pavlovian conditioned freezing [65], while mice with brain-wide deletion of ADK developed spontaneous seizures and profound deficits in hippocampus-dependent learning and memory [66]. Humans with ADK deficiency showed hepatic encephalopathy, developmental delay, and cognitive impairment [53,54]. Many actions of adenosine are mediated by adenosine receptors [23] while others are receptor-independent [29]. Indeed, A2AR antagonists can attenuate neurological symptoms in ADK deficiency [66] while intracellular adenosine acting on the transmethylation pathway, plays a role in the modulation of epileptogenesis [67]. Transmethylation reactions require S-adenosylmethionine as donor of the methyl group and generate S-adenosylhomocysteine (SAH) as a product, which is then further converted into adenosine and homocysteine by SAH hydrolase (SAHH). ADK deficiency leads to accumulation of adenosine, which reverses the SAHH reaction, leading to high levels of SAH, which inhibits the transmethylation reactions [68]. Spontaneous recurrent seizures in murine models were associated with disruption of adenosine homeostasis (increased ADK and reduced adenosine), increased DNA methyltransferase activity and increased hippocampal DNA methylation [67]. Intraventricular implantation of adenosine-releasing polymers restored methylation to control levels and reduced seizure activity even after cessation of adenosine release from the polymers. In addition, DNA methyl transferase inhibition reduced seizure susceptibility and epilepsy acquisition [67]. Interestingly, genetic variants of ADK have been associated with the development of post-traumatic epilepsy in humans [69]. Therefore, changes in adenosine metabolism, such as those triggered by pathological hyperexpression of ADK or by genetic mutations, could be biomarkers for epileptogenesis and also therapeutic targets to prevent epilepsy. 

Other studies have demonstrated that ADK determines the degree of brain injury after ischemic stroke in mice. When ischemia has been induced in transgenic mice with increased ADK expression in striatum and reduced ADK expression in cortical forebrain, the infarct volume was increased in the striatum and decreased in the cortex compared with WT controls [49].

The neuroprotective effects of adenosine suggest its use as a potential therapeutic agent for various brain disorders. However, systemic application of adenosine is hampered by cardiovascular side effects. In order to improve local delivery and raise the level of adenosine in CNS, adenosine releasing polymers and cell-mediated cell therapy have been proposed. Indeed, human neuroepithelial stem cells with *ADK* gene knockout were able to differentiate in cells exhibiting higher release of adenosine compared to control cells [70].

### 3.3. ADK and ADA in Glioma

Recent evidence indicates that ADK and ADA levels are related to glioma progression. ADK was detected in the cytoplasm as well as in the nuclei of cells obtained from gliomas. ADA and ADK levels were upregulated in patients with Grade II and Grade III gliomas compared to control subjects [57,58]. ADK and ADA, by regulating adenosine concentration, might also play a role in tumor growth and apoptotic cell death in gliomas, modulating proliferation of glial and endothelial cells [71,72,73]. Extracellular adenosine reduced viability in rat glioma cells [71,72] and ADA reverted this effect [72]. Therefore, the higher expression of both enzymes is expected to increase tumor growth. However, the in vivo effect may depend on the expression of the different adenosine receptors in tumoral and peritumoral cells as well as on the oxygen levels. Interestingly, hypoxic niches of glioblastoma induced tumorigenic properties of a small cell subpopulation called glioblastoma stem-like cells which generated extracellular adenosine and were able to differentiate into endothelial cells [74]. The blockade of A3AR reduced both the differentiation under hypoxia and blood vessel formation in vivo [73]. ADA and ADK expression was upregulated in peritumoral tissues derived from patients with epilepsy compared to those without epilepsy [57,58], whereas the number of ADA-positive or ADK-positive cells in tumor tissues was similar between glioma patients with and without epilepsy [58]. ADA activity increased following induction of seizures in zebrafish, while pretreatment with anti-epileptic drugs before the convulsant prevented the stimulatory effect on ADA activity [75]. Therefore, it appears that increased ADA and ADK may reduce adenosine levels, decrease its inhibitory activity and lead to epileptogenesis and progression of epilepsy in glioma patients. 

One side effect of chemotherapy is neuropathic pain, that might be promoted by enhanced spinal ADK levels through a mechanism dependent on astrocytes [76]. Chemotherapeutic agents such as oxaliplatin caused ADK hyperexpression in reactive astrocytes and reduced adenosine signaling at the A3AR subtype within the spinal cord in rodents. Dysregulation of ADK and A3AR signaling was found to be associated with increased proinflammatory and neuroexcitatory interleukin-1β expression and inflammasome activation, while the application of a selective A3AR agonist attenuated the production of the inflammatory cytokine IL-1β and increased the release of the anti-inflammatory IL-10 [76]. 

### 3.4. ADA

ADA catalyzes the deamination of adenosine and deoxyadenosine. Mutations in the *ADA* gene are among the most common causes for severe combined immunodeficiency (SCID). In the absence of ADA activity, deoxyadenosine accumulates in extracellular compartments and within cells, where it is converted by deoxycytidine kinase and/or ADK to deoxyadenosine monophosphate, which in turn is converted to deoxyadenosine triphosphate (dATP). Intracellular dATP might generate DNA strand breaks, inhibit ribonucleotide reductase, thereby impairing DNA synthesis and repair, induce apoptosis in developing thymocytes and interfere with terminal deoxynucleotidyl transferase activity [77,78]. Additionally, deoxyadenosine inactivates SAHH, leading to accumulation of SAH (see above) and inhibits the transmethylation reactions necessary for effective lymphocyte activation. The accumulation of deoxyadenosine and dATP in lymphocytes is considered as the primary cause of lymphotoxicity.

Besides immune effects, ADA-SCID patients show skeletal, hepatic, renal, and lung alterations, as well as neurological abnormalities and behavioral impairments. They include reduced verbal expression, learning disability, hyperactivity, attention deficit, seizures, and hearing deficits [79,80,81]. Interestingly, reduced ADA activity has also been found in the serum of autistic children [82], in association with a polymorphism in the ADA gene [83]. This rare low-activity polymorphism was more likely found in children with mild mental retardation of unknown causes, when compared to both healthy controls and children with moderate to severe mental retardation of known causes [84].

Although bone marrow transplant, PEG-ADA treatment or hematopoietic stem cell gene therapy are able to improve the immunological/metabolic deficits, they cannot completely prevent the onset or resolve pre-existing neurological defects [85,86,87,88]. It is difficult to ascertain whether the neurological impairments are exclusively determined by the lack of ADA or are also a consequence of the complications from infections at early life. It is thought that different types of stress in early life may particularly alter neuro-immune development with psychiatric consequences [89]. Adenosine and deoxyadenosine accumulate when there is a deficit of ADA, and they might be responsible for the alterations in the nervous system. Adenosine acts as a neuromodulator through a family of purinergic G-protein-coupled receptors (A1R, A2AR, A2BR, and A3R), while ATP binds to P2XR and P2YR (see introduction).

Recently, the extent of correction of neurological deficits after PEG-ADA treatment has been studied in an animal model of ADA-SCID, the *Ada*−/− mouse [88], which showed many features associated with ADA deficiency in humans, including systemic metabolic alterations and immunodeficiency [90]. Brain size of *Ada*−/− mice was slightly reduced compared to *Ada*+/+. Brains of 3-week-old *Ada*−/− mice showed ventriculomegaly but not myelination alterations or neuronal loss [88]. In *Ada*−/− mice, ADA activity was undetectable, and adenosine increased in total brain extracts from birth until their death, while adenosine levels in *Ada*+/+ mice remained low. Lack of *Ada* led to alterations in explorative behavior, increased anxiety-like behavior, and reduced pain sensitivity without modifying sensory or motor development at 15th postnatal day. *Adora2a*−/− mice were hypoalgesic [91], suggesting that *Ada*−/− might have defects in A2AR signaling. Indeed, the level of A2AR in *Ada−/−* and in PEG-ADA-treated mice was reduced compared to *Ada+/+* brains. Pain sensitivity showed a tendency to decrease after PEG-ADA treatment, without reaching wild type levels [88]. ADA activity was not detected in the brain of PEG-ADA-treated *Ada−/−* mice, likely because PEG-ADA cannot cross the blood–brain barrier. Adenosine metabolite levels in the brain of PEG-ADA-treated mice were 10-fold lower than in untreated mice, but remained 3-fold higher than in wild type. The authors hypothesized that brain adenosine metabolites could diffuse out of the brain and could be detoxified peripherally by PEG-ADA. This could result in reduced adenosine levels in the brain. Interestingly, the enzyme replacement therapy corrected the ventriculomegaly but not the observed abnormalities in exploration and anxiety-like behavior. Therefore, some of the characteristics of the *Ada−/−* mice could be due to alterations in the adenosine receptors [88]. 

### 3.5. ADA and Adenosine Receptors

Accumulating evidence suggests that ADA and adenosine receptors interact at the cell surface [92], probably increasing receptor sensibility to adenosine. Indeed, ADA binding affected the quaternary structure of A2AR [93] and A1R [94] and increased both agonist and antagonist binding. Under normal conditions, endogenous adenosine modulated colonic motility via A2BR located in the neuromuscular compartment. In the presence of bowel inflammation, this inhibitory control was impaired due to the link between A2BR and ADA, which when catabolizing adenosine, prevented A2BR activation [95]. ADA acts not only as a catalyst, but also as a costimulator, an allosteric modulator and a cell-to-cell connector [96]. Indeed, there is also evidence of the formation of trimeric complexes dipeptidyl peptidase IV (CD26)-ADA-A2AR involving two cells. ADA could bridge T-cells (expressing CD26) and dendritic cells (expressing A2AR) [96], but it is still unknown if this role is also played in the nervous system and whether the ADA deficiency could affect the connection between nervous cells. CD26 has been associated with a variety of pathologies, including tumors, but no correlation between CD26 expression in gliomas and malignancy has been found [97].

### 3.6. ADA Inhibition and Apoptosis

It is known that accumulation of deoxyadenosine and/or adenosine can cause apoptotic death in sympathetic neurons and adrenal chromaffin cells [98,99], in human astrocytoma and neuroblastoma cells [100,101,102]. Adenosine also induced death of embryonic stem cell-derived motor neurons in culture [103]. The effect of adenosine and deoxyadenosine may depend on the developmental stage of the cell. In cultured chick sympathetic neurons, adenosine was lethal at earlier stages of development but not later (i.e., when added to the culture from the time of plating up to 16 h). 

Deoxycoformycin (dCF), a powerful inhibitor of ADA [104], has been used alone or in combination with other drugs for the treatment of several types of lymphocytic leukemia [105,106,107,108]. The combination of deoxyadenosine and dCF is toxic for several cell lines of tumoral origin such as rat hepatoma cells [109] and human colon carcinoma cell lines LoVo and HT29 [110,111,112]. The treatment with deoxyadenosine and dCF in combination induced apoptosis in human astrocytoma and neuroblastoma cell lines [100,101,102], but the underlying mechanisms seem to be different. In astrocytoma cells, a reduction in the production of lactate preceded the effect of deoxyadenosine and dCF on cell viability, suggesting a decreased glycolytic capacity, while neuroblastoma cells did not show changes on glycolytic capacity. Both cell lines showed a decrease in mitochondrial reactive oxygen species production probably due to an impairment in mitochondrial function. The observed increase in mitochondrial mass could possibly help to cope with the reduction in mitochondrial activity. In both cell lines, deoxyadenosine must be phosphorylated in order to exert its cytotoxic effect; however, a decrease in the energy charge was observed in astrocytoma but not in neuroblastoma cells. Therefore, it appears that one of the roles of ADA is to avoid the accumulation of the potentially toxic adenosine and/or deoxyadenosine. This action can be useful in fighting tumors, since ADA inhibition could lead to increased cell death, but at the same time, decrease of ADA activity might increase the risk of neurological dysfunction, including cognitive deficits. Recently, another contribution of ADA inhibition in the suppression of tumor progression has been highlighted. It has been demonstrated that dCF suppresses growth of 4T1 murine breast cancer in vivo, and in vitro experiments have shown that dCF reduces migration, invasion and adhesion of 4T1 cells and the involvement of A2AR and A3R activation in these processes [113].

## 4. Hypoxanthine-Guanine Phosphoribosyltransferase (HPRT)

### 4.1. HPRT and LND

LND is a severe X-linked hereditary disorder caused by the lack of the enzyme HPRT. The metabolic symptoms of this disorder are hyperuricemia leading to gout and impaired kidney functions, accompanied by neuropsychiatric problems such as dystonia, spasticity, and self-injurious behavior, the hallmark feature of LND [114]. The overproduction of uric acid can be explained by the conversion of unrecycled guanine and hypoxanthine into uric acid and the increase in the rate of purine de novo synthesis, which has been longly ascribed to the accumulation of PRPP and reduced inhibition by end-products [115]. While the treatment with allopurinol, an inhibitor of XOD, reduces plasma concentrations of uric acid, no effects on neurological symptoms are observed [115]. So far, the link between the metabolic defect and the neurological manifestations remains largely unknown. There is strong evidence that the neurological impairments in LND are mainly due to the effects of HPRT deficiency on both the development of dopaminergic neurons [116] and on the modulation of GABAergic and glutamatergic neurotransmission [115]. Positron-emission tomography of brains of LND patients revealed decreased dopamine production and storage [117] and a marked reduction in dopamine transporters [118]. Despite these earlier reports which indicate that LND primarily involves the basal ganglia, later on, magnetic resonance imaging studies revealed substantial white matter volume reduction in LND patients in brain regions which is consistent with the neurobehavioral phenotype of LND patients, thus arguing for more complex mechanisms beyond the basal ganglia [119].

### 4.2. HPRT Deficiency and the Accumulation of Potentially Toxic Metabolites

A huge amount of work has been done to find the molecular link between the metabolic defect and neuronal development in LND patients. It has been hypothesized that the excess hypoxanthine accumulating as a consequence of HPRT deficiency triggers the neurological abnormalities [120]. Indeed, primary astroglia cultures from HPRT-deficient transgenic mice [121] and a rat neuroma cell line deficient of HPRT [122], both used as an in vitro model of LND, showed a 15-fold increase in extracellular hypoxanthine, due to the lack of recycling to IMP. A significant accumulation of hypoxanthine was observed also in human neuroblastoma HPRT-deficient sublines [123] and in cultures of fibroblasts obtained by LND patients [124]. In a rat neuroblastoma B103 cell line and its HPRT-deficient mutant (B103-4C), hypoxanthine significantly increased the proliferation of both cell lines with a greater effect on the mutant cells. Also, differentiation was affected by hypoxanthine, but while an enhancement was observed in control cells, a decrease was reported for the mutant cells [125]. The authors suggest that exposure to hypoxanthine during fetal or perinatal development of the nervous system of LND subjects, through a still unknown mechanism, probably involving the binding to benzodiazepine receptor [115,126], may cause abnormal arborization of dopaminergic neurons, which has been suggested to be responsible for the symptoms of LND [127]. A deregulation of genes involved in early neuronal development by hypoxanthine has been reported in a human embryonic carcinoma neurogenesis model (NT2/D1) [128]. The authors report that, during retinoic acid-induced differentiation, excess hypoxanthine, through unidentified mechanisms, significantly increased the expression of genes associated with neural development such as wingless-type MMTV integration site family, member 4 (WNT4), belonging to the Wnt/β-catenin pathway, and engrailed homeobox 1, a transcription factor known to play a key role in the specialization and survival of dopamine neurons. Hypoxanthine appears also to enhance the expression of tyrosine hydroxylase, the rate-limiting enzyme in dopamine synthesis, and type 1 dopamine receptors A2AR and 5-hydroxytryptamine (serotonin) receptor 7, whose hyperexpression characterize early neurodevelopmental processes [128]. Similarly, an imbalance in adenosine, dopamine, and serotonin receptors has been reported in lymphocytes of LND patients [129]. This supports the hypothesis that the neurological impairments of LND patients may be related to an imbalance of more than one neurotransmitter. In fact, adenosine, dopamine, and serotonin receptors, belonging to the G-protein coupled family, seem to be integrated through intermembrane receptor–receptor interactions, and adenosine receptor disturbance may be followed by alterations in dopamine and serotonin receptor [129]. Also, a hypothesized deficiency of guanine-based purines has been invoked as responsible for the neurological manifestations in LND [115]. Guanosine is recognized as an important modulator of glutamatergic neurotransmission, promoting glial re-uptake of L-glutamate [130]. Deutsch et al. [115] hypothesized that the lack of HPRT in LND does not allow the salvage of guanine-based nucleotides, thus leading to a depletion of guanine-based purines in areas critical to normal synaptic neurotransmission, including the area of glutamatergic synapses. Indeed, in neurons, the rate of the degradative deamination of guanine to xanthine by guanase is significantly greater than its incorporation into nucleotides by HPRT [131], therefore, owing to the lack of salvage of guanine in LND, guanine appears to be channeled towards degradation.

Using peripheral blood lymphocytes obtained from controls and LND patients, Torres et al. [132] demonstrated that HPRT deficiency causes a reduction in adenosine uptake, and this reduction is more pronounced if hypoxanthine is present in the culture medium. This could be attributed to dysfunction of the adenosine equilibrative transporters, and in particular, the NBTI-insensitive (ENT2) transporters appeared to be predominantly affected, hypoxanthine being a competitive inhibitor of adenosine transport [132,133]. Inhibitors of adenosine transport have been reported to increase extracellular cerebral adenosine levels [134,135] by inhibiting entry of the nucleoside into cells which actively metabolize adenosine through ADA and/or ADK [136]. Therefore, it is assumed that inhibition of adenosine uptake, due to extracellular hypoxanthine accumulating in LND patients, causes an extracellular accumulation of adenosine with enhanced stimulation of adenosine receptors [120]. While adenosine in CNS inhibits neurotransmitter release by the A1 receptor, the nucleoside can also induce opposite effects through the A2AR. These receptor subtypes are expressed in different areas of the developing brain, and a correct integration between excitatory and inhibitory stimuli is regulated by their timely recruitment. In LND, excitatory A2AR-mediated signaling is likely predominant compared to A1R-mediated inhibitory processes [25]. Recently, it has been reported that hypoxanthine infusion in striatum of young Wistar rats increases neuroinflammatory parameters perhaps through oxidative misbalance [137] and induces neuroenergetic impairment resulting in ATP depletion which leads to mitochondrial dysfunctions and cell death by apoptosis [138]. Therefore, the authors suggest that these processes may be involved, at least in part, in the pathogenesis of the neurological disorders of LND patients.

An accumulation of 5-aminoimidazole-4-carboxamide ribonucleotide (AICAR) (also known as ZMP) has been reported in LND patients [139] and in a patient lacking AICAR formyltransferase/IMP cyclohydrolase (ATIC), an enzyme belonging to the de novo purine synthesis, which catalyzes the two-step conversion of ZMP into IMP. The ATIC-deficient patient presented devastating neurological symptoms, including seizures, developmental delay, hypotonia, autistic features and brain anomalies [140]. ZMP is an AMP mimetic known to activate the AMP-activated protein kinase (AMPK) [141], and the treatment of undifferentiated neuroblastoma cells with AICA-riboside activated AMPK and exerted a neurotoxic action through the apoptotic program [142], possibly triggered by the prolonged and uncontrolled AMPK activation. In this regard, López [143] hypothesized that ZMP may be the toxic compound in LND. Indeed, the prolonged activation of AMPK may exert noxious effect on dopamine synthesis [144], cell viability and morphology. 

### 4.3. HPRT and the Formation of Purinosome

After the discovery of purinosome [4], many attempts have been made in order to identify the signal responsible for the aggregation/disaggregation of the de novo purine synthesis enzymes. Chan et al. [145] demonstrated that the assembly/disassembly of the purinosome depends on the cell cycle and correlates with cellular demands for purine biosynthesis encountered during the cell cycle. In particular, the number of purinosome-containing cells peaks in G1 phase in HeLa cells grown and maintained in a purine-depleted condition. The purine demand decrease correlates with the decrease in purinosome-positive cells in S and G2/M phases of cell cycle. HPRT-deficient fibroblasts also exhibited the greatest purinosome formation in G1 phase, however high levels of purinosomes were also observed in the S and G2/M phases [145]. Accordingly, Fu et al. [124], using cultures of fibroblasts obtained from HPRT-deficient patients and normal healthy controls grown under purine-rich conditions, demonstrated that the formation of purinosomes was significantly greater in fibroblasts of LND patients, suggesting that the lack of HPRT activates the de novo synthesis, independently of the presence of preformed purines. The authors suggest that the increase of de novo purine synthesis could compensate for the loss of purine recycling. However, the excreted uric acid largely exceeds the amount of purine nucleotides necessary to compensate for the lack of purine recycling, indicating a dysregulation of the purine de novo synthesis. Using a synchronized human colon carcinoma cell line, Fridman et al. [3] showed that purine nucleotide synthesis by both the de novo and salvage pathways increases markedly when cells progress from G1 into the S-phase, and that this increase is driven by a raise in PRPP concentration. Since the increased de novo synthesis correlates with the formation of purinosome [145], it is conceivable that PRPP, which accumulates in case of HPRT deficiency, favors the formation of purinosome, which could explain the dysregulation of this synthetic pathway in LND patients.

### 4.4. HPRT and the Purinergic Signaling

While studying the high-affinity GTPase activity of G-proteins in membrane from primary human skin fibroblasts, rat B103 neuroblastoma cells and mouse Neuro-2a neuroblastoma cells, Pinto et al. [146] unexpectedly found that the membrane low-affinity nucleoside 5-triphosphatase activity (NTPase) was decreased up to 7-fold in HPRT deficiency. This first report, based on the measurement of enzyme activity, was unable to reveal the molecular identity of the involved NTPases. Therefore, RT-PCR and restriction enzyme digestion of amplified cDNA fragments were used to evaluate the expression, in B103 and Neuro2a cells, of different isoforms of ecto-nucleoside 5′-triphosphate diphosphohydrolases (NTPDases), a likely candidate enzyme family responsible for the altered NTPase activity in HPRT deficiency [147]. Collectively, the expression of NTPDase isoforms in HPRT deficiency depends on the specific cell type and species studied, so that, depending on the overall change in NTPDase isoenzyme expression and the specific activities of isoenzymes, the net result may be an increase or a decrease in NTPDase activity [147]. Since extracellular ATP regulates cell function through P2XRs, belonging to the class of ligand-gated ion channels, and through P2YRs, belonging to the class of G-protein coupled receptors, the hypothesis of an association between HPRT deficiency and abnormalities in P2XR and P2YR-mediated signaling has been tested in rat B103 neuroblastoma cells [148]. The measurement of the effect of various nucleotides on both the influx of Ca^2+^ across the plasma membrane and the mobilization of Ca^2+^ from intracellular stores, associated with quantitative RT-PCR studies, revealed that two ligand-gated ion channel subtypes (P2X2R and P2X4R), and five G-protein coupled receptors (P2Y2R, P2Y4R, P2Y12R, P2Y13R and P2Y14R) are downregulated in HPRT deficiency [148]. Therefore, an altered purinergic Ca^2+^ signaling in a HPRT-deficient cell model for LND has been reported. Later on, Mastrangelo et al. [149] demonstrated a downregulation of P2Y1R expression in HPRT-knockdown human induced pluripotent stem (iPS) and embryonic stem (ES) cells. HPRT-deficient iPS cells show highly impaired CREB phosphorylation and constitutively activated ERK1/2 and insensitivity to ATP. ERK proteins belong to the MAPK family, which responds to growth factors and regulates proliferation and differentiation [150]. Therefore, HPRT may play a role in determining aspects of neurogenesis and neurodevelopment during embryogenesis by regulating P2Y1R purinergic signaling. HPRT deficiency may hamper the function of embryonic and developing CNS cells, thus contributing to the neuropathology of LND [149]. In fact, using HPRT-deficient ES cells as model and global transcriptomic characterization as method, it has been demonstrated that HPRT-knockdown causes a switch from neuronal to glial gene expression [151]. Even though the exact nature of the responsible mechanisms has not been definitively established, a possible candidate could be the aberrant expression of *Sox2*, a gene vital for stem cell pluripotency and for the neuronal/glial cell fate decision [152]. The persistent and increasing expression of *Sox2* in the differentiating HPRT-deficient murine ES cells appears to be consistent with the impaired neurogenesis typical of HPRT deficiency [151]. Moreover, HPRT-deficiency causes dysregulated expression of key genes such as achaete-scute family bHLH transcription factor 1 (*Ascl1*), forkhead box P1 (*Foxp1*), and transcription factor B-cell leukemia 11b (*Bcl11b*), essential for striatal patterning, and the gene encoding dopamine- and cAMP-regulated phosphoprotein 32 (*Darpp-32*) in immortalized mouse striatal neural stem cells. On the other hand, HPRT-deficiency upregulates the expression of the brain-derived neurotrophic factor (BDNF)/TrkB pathway, and thereby confers protection to HPRT-deficient striatal cells against reactive oxygen species-mediated cell death [153]. The authors suggest that the purine metabolic defect caused by HPRT-deficiency, while providing neuroprotection to striatal neurons, affects key genes and signaling pathways that may underlie the neuropathogenesis of LND [153]. In addition, HPRT deficiency in murine ES cells dysregulates many cellular functions controlling cell cycle and proliferation, RNA metabolism, DNA replication and repair, protein synthesis, and others [151]. Also, an increased expression of the miR181a in HPRT-deficient human dopaminergic SH-SY5Y neuroblastoma cells has been identified, which could lead to aberrant expression of many target genes involved in embryonic development of CNS [154]. Therefore, it is conceivable that neural aberrations of LND result from combinatorial and multigenic defects. Intriguingly, many of the dysregulated genes during neurogenesis of HPRT-deficient murine ES cells are also found aberrantly expressed in AD and other CNS disorders [155]. This suggests that, even though the gene expression dysregulations in AD are not resulting directly from aberrant expression of HPRT, disturbances of purine metabolism and purinergic signaling are likely to contribute to dysfunction in AD and possibly other CNS disorders.

### 4.5. HPRT and Cancer

In 1990, our research group reported that HPRT activity was significantly higher in tissue samples obtained from patients operated upon for intestinal and breast cancer with respect to peritumoral tissues [156]. The increased activity appears to confer selective growth advantage to cancer cells, favoring a metabolic flux towards purine mononucleotides, which can be used as precursors for the synthesis of nucleic acids. In addition, a progressive and statistically significant increase in HPRT activity was observed with clinical parameters of human colon carcinoma diffusion [157]. Recently, several research groups resumed this topic, postulating an emerging role for HPRT in cancer. Müller et al. [158] using quantitative PCR, found that HPRT was detectable in cultured human MDA-MB-231 breast carcinoma cells, primary tumors, and tumor-infiltrated lungs of SCID-mice injected with human MDA-MB-231 breast carcinoma cells, but undetectable in normal mouse lungs, and that the human *HPRT* mRNA content in lungs of mice correlated with the size of the tumor. More recently, using immunohistochemistry staining, the level of HPRT has been evaluated in normal and malignant tissues from human lung, breast, colon and prostate [159]. Although a significant variability within patients with regard to the relative expression of HPRT both in normal and malignant tissues has been observed, an overall trend showing upregulation of HPRT in cancerous tissues has been reported. With the exception of lung cancer, the authors claim that the upregulation of HPRT is independent of cancer grade or stage, and they hypothesize that HPRT could be utilized as an early biomarker because it appears to be upregulated in cancer regardless of stage [159]. A surface expression of HPRT has been reported in lung cancer cell lines, although the reason for this external presentation is presently unknown [160]. As HPRT is a potential cancer-associated antigen, the authors hypothesize that HPRT could become a target for emerging immunotherapies designed to attack cancer cells which display unique surface proteins [161].

## 5. Xanthine Oxidase (XOD)

### 5.1. XOD and Xanthine Dehydrogenase (XDH) 

Hypoxanthine and xanthine oxidation to uric acid can be catalyzed by two different activities: XDH and XOD, both expressed by the same protein [162]. During the dehydrogenase reaction electrons from substrates are transferred to FAD and eventually to NAD^+^ which is reduced to NADH, while in the oxidase reaction, the reduced FAD donates electrons to oxygen, thus producing hydrogen peroxide and superoxide. XDH can be converted to XOD by disulfide formation or proteolysis [163]. It is highly possible that in certain pathological conditions this transformation occurs in vivo, but it is extremely difficult to know the relative concentration of the two forms in living organisms and cells [162]. However, it would be very interesting to have an idea about the presence of the two forms, because both of them generate uric acid with antioxidant activity, but XOD generates also activated oxygen species. As a consequence, enzyme inhibitors can cause a decrease of antioxidant defense that can be dangerous for brain function [31], but can also decrease the oxygen radical generation, protecting cells, and organs expressing XOD from oxidative stress [164].

### 5.2. XOD and Uric Acid

Uric acid is the metabolic end product of purine catabolism in humans. Generally, in other mammals, uric acid is transformed into allantoin which is a more soluble compound. Catabolism of purine compounds occurs in all organs of the human body, but the final step of xanthine and hypoxanthine oxidation into uric acid occurs only in tissues expressing XOD, mainly liver and small intestine [165]. A considerable XOD activity has also been measured in mammary glands where it appears to be involved in the correct formation of the envelop of milk fat droplets [166]. Because of the high level found in colostrum and early milk, XOD activity has long been considered an important host defense molecule in the intestine of breastfed infant [167].

Humans are unable to metabolize uric acid because of a mutation in the uricase gene, following the food shortage and global cooling 15 million years ago, which presumably yields a survival advantage [168]. The consequence of uricase inactivation is the appearance of urate levels that are much higher in humans in comparison to other mammals. It has been demonstrated that uric acid is a potent antioxidant accounting for approximately 50% of the antioxidant power in humans [169]. The loss of uricase in hominids, along with the fact that in humans 90% of uric acid filtered by the kidneys is reabsorbed, suggests that uric acid is a somehow beneficial compound and not just a mere waste product. Indeed, uric acid is no longer considered biologically inert since it acts as a pro- and antioxidant, neuroprotector, neurostimulant, and activator of the immune response and inflammation [170]. Nevertheless, hyperuricemia is associated with multiple diseases in humans and this finding points to the deleterious effects of high concentrations of urate [32]. Consequently, the manipulation of serum uric acid levels has become a popular strategy for the treatment of a considerably high variety of diseases, and the research on the molecular links between uric acid and several pathologies and particularly neurodegenerative disorders has been pushed forward [171]. The amount of plasma urate depends on the dietary intake of purines, mainly degraded in the intestinal mucosa, and on the rate of their biosynthesis and excretion [31]. The major regulatory system for the homeostasis of plasma urate depends on a four-component renal transport system which involves glomerular filtration, reabsorption, secretion, and post-secretory reabsorption [172]. From several studies a normal range of blood uric acid concentration can be defined between 120 and 380 µM depending on gender and age [173]. 

### 5.3. Pathologies Associated with High Uricemia

Hyperuricemia has been implicated in the etiology of several diseases such as diabetes, chronic kidney disease, coronary heart disease, and many others [174]. An increase of serum uric acid can be caused by a defect in the proteins implied in its homeostasis, such as those involved in renal urate clearance [175], or as a consequence of the assumption of food such as fatty meat, organ meat, seafood, or high amount of fructose [176,177]. High levels of uric acid have been described also as a consequence of an increase of the rate of purine synthesis, turnover, or catabolism (see above in this review). A number of reviews and papers discuss the correlation between uric acid level and the pathogenesis of several diseases [31]. Recently, XOD and uric acid have been reported to be involved in the pathogenesis of the metabolic syndrome and insulin resistance. However, no clear explanation has been advanced for the adverse effect exerted by XOD and uric acid [164,174]. Several papers indicate a strong association between high concentration of circulating uric acid and inflammation and cancer [178,179,180]. It has been hypothesized that uric acid plays an important role as a signaling molecule mediating the inflammatory effects of hyperuricemia in adipocytes and leukocytes, and as a promoter of tumor cell proliferation, migration, and survival [181]. Therefore, managing uric acid levels may be relevant for the improvement of treatment strategies in patients in which hyperuricemic disorders are associated with tumors [181]. 

Inhibitors of XOD have been widely utilized to contain the dangerous effects of uric acid accumulation, because they cause a decrease of circulating uric acid concentration and an increase of xanthine and hypoxanthine [182]. The effects of high hypoxanthine concentration, associated both with XOD inhibition and HPRT deficiency, are described above in this review, while the possible involvement of hypouricemia in the etiology of several neurodegenerative diseases will be discussed in the following section.

### 5.4. Pathologies Associated with Low Uricemia

Uric acid is maintained through a complicated homeostatic mechanism, at a very high concentration in blood, close to the limit of its solubility. As mentioned before, uric acid is potent antioxidant, but it has been claimed that it is also a stimulator of the cerebral cortex [183]. The oldest hypotheses of an influence of uric acid concentration on intelligence were based on the similarity of the uric acid structure with some brain stimulants such as caffeine [184]. Since then several authors have found significant positive correlation between uric acid concentration and higher intelligence in children and adults [185,186]. Furthermore, a positive correlation exists between gout and intelligence [183]. On the bases of these findings, a role in neuroprotection was hypothesized for uric acid, reinforced by the observation that gout and multiple sclerosis are mutually exclusive [31]. Recently, many authors suggested that the neuroprotective action of uric acid was due to its antioxidant activity. In fact, the brain is very vulnerable to oxidative damage and requires a very efficient antioxidant defense since it has a very high metabolic rate and a high presence of unsaturated fatty acid in its membrane [187]. A metabolomic study pointed out that uric acid decreased in serum of patients suffering from AD, Parkinson’s disease, and amyotrophic lateral sclerosis (ALS) [188]. The role played by uric acid in serum and cerebrospinal fluid was mostly studied in Parkinson’s disease, where oxidative stress plays a major role [189]. It was found that, not only uric acid inversely correlated with the risk of developing the disease in the general population [190], but also that uric acid level correlated with certain motor and non-motor disturbances [191]. In particular, patients with higher serum uric acid were more likely to manifest resting tremor and less likely to develop other non-motor disturbances such as mild cognitive impairment. An inverse association was also found in patients with fatigue, the same observation was also reported in more severe form of fatigue such as in acute ischemic stroke and chronic fatigue syndrome [192,193,194]. In a Parkinson’s disease mouse model, it was demonstrated that uric acid administration resulted in a significant neuroprotection for dopaminergic neurons probably through Nrf2-ARE-induced inhibition of oxidative damage and neuroinflammation [195]. ALS patients were shown to have lower serum uric acid levels than healthy individuals by several studies [188,196]. The decreased uric acid levels were correlated with the rate of disease progression, further demonstrating the possible role of oxidative stress in the induction and propagation of the disease, and the possible therapeutic role of uric acid administration [196]. Curiously, it was also recently demonstrated that inhibition of XOD in ALS animal model, with non-purine analog drugs, had a protective effect on motoneurons [197]. Further studies are necessary to unravel the causal linkage between uric acid concentration and neurodegeneration, and in particular, in our opinion, it would be necessary to investigate on the cause of the low uric acid concentration associated with neurodegeneration. In some cases, this scarcity may be the consequence of a low purine turnover. In this light, the paradoxical effect of XOD inhibition in ALS could be explained as a mean to increase circulating hypoxanthine and guanine for purine nucleotide synthesis through salvage pathway, necessary to sustain neuron energy supply. The general neuroprotective role exerted by uric acid was also described in ischemic stroke; in fact clinical epidemiological studies demonstrated that ischemic stroke patients with higher serum urate levels had better clinical outcomes upon hospital discharge [198]. On the bases of all the observations listed above regarding the neuroprotective effects exerted by uric acid, clinical trials of uric acid administration in acute ischemic stroke, as well as in Parkinson’s disease were set up, demonstrating a certain degree of efficacy more pronounced in women than in men [199,200]. Since women have substantially lower serum uric acid levels, this observation is attractive and requires further investigation. Alternatively, inosine, a uric acid precursor, has been found to improve outcomes, both in animal models and patients in a number of neurological disorders [171,201,202]. In this case however, the effect exerted could be more difficult to decipher because inosine is not only an uric acid precursor, but can increase purine supply for nucleotide synthesis, ribose moiety for PRPP synthesis or energy supply and finally can also engage adenosine receptors [203]. 

## 6. Concluding Remarks 

Owing to the multitude of biological processes in which purines are involved, complex enzymatic machinery has been built up in order to respond to the varying demands of the organism. Therefore, it is not surprising that alterations in the activity of key enzymes of purine metabolism may lead to severe pathological manifestations. Nevertheless, the consequences of enzyme dysfunctions are sometimes surprising, unexpected and difficult to understand at the molecular level. Experimental evidence suggests that disturbances of purine metabolism are likely to contribute to dysfunction in AD and possibly other CNS disorders. An association between purine metabolism dysfunctions and tumor progression has also been reported. In fact, tumor cells express a greater amount of cN-II, which appears to confer resistance to several purine prodrugs commonly used in chemotherapy. Also HPRT is upregulated in several solid tumors, thus favoring a metabolic flux towards purine mononucleotides, used as precursors for the synthesis of nucleic acids. As a consequence, a selective growth advantage is given to cancer cells. ADA and ADK are upregulated in higher grade gliomas but the relevance for tumor progression requires further research. Some metabolic manifestations of purine enzyme dysfunctions that are clearly related to substrate accumulation or lack of product are often associated with other manifestations, mainly neurological, that are not so clearly and directly related to the enzyme dysfunctions. As an example, a complete lack of HPRT activity causes uric acid accumulation which leads to kidney failure that can be reversed by XOD inhibition, but also a very complex and severe neurological dysfunction that despite 60 years of studies and several very interesting reports, still waits for a clear explanation. In ADA-deficient patients, a deoxyadenosine accumulation has been observed, which leads to an increase of intracellular dATP concentration that acts as a ribonucleotide reductase inhibitor and is lethal for cells proliferating at high rate. The same patients, despite a substitutive ADA-PEG therapy, develop a variable degree of neurological and cognitive impairments, possibly ascribable to an adverse effect exerted by adenosine accumulation on the developing brain. However, the molecular basis of this effect still needs to be clearly addressed. Although the study of the effects caused by cN-II mutations is still at the beginning, it is attractive that the only hereditary disease linked to cN-II mutation, so far ascertained, is spastic paraplegia, a multifactorial disease due to dysfunction of long axons in the spinal cord. Many old reports indicate a positive correlation between high uricemia and intelligence; the consequence of these observations might bring the reader to the conclusion that middle-aged men are more intelligent than young-men and women, which is far from true. High uricemia has been also associated to several diseases such as metabolic syndrome, diabetes, and cancer. The nature of this relationship is matter of debate but the results are clear and point to an adverse effect exerted by high concentrations of uric acid. Indeed, allopurinol and other more recently commercialized XOD inhibitors are among the most frequently prescribed drugs in the highly developed countries. The consequence of XOD inhibition is a decrease of uric acid and an increase of xanthine and hypoxanthine serum concentrations. However, as highlighted in the present review, high concentrations of hypoxanthine may interfere with the purinergic signaling, and low uric acid concentrations have been associated with neurodegenerative diseases.

Overall, many neurological aspects of a variety of diseases of unknown mechanism might rely on a deficit or deregulation of the enzymes involved in purine metabolism. Therefore, we think that the diagnosis of these neurological diseases could benefit from the measurement of the activities of purine metabolizing enzymes, paving the way for new effective therapeutic approaches.

## Abbreviations

A1R	adenosine receptor subtype 1
A2R	adenosine receptor subtype 2
A3R	adenosine receptor subtype 3
AD	Alzheimer’s disease
ADA	adenosine deaminase
ADK	adenosine kinase
AICAR	5-aminoimidazole-4-carboxamide ribonucleotide
ALL	acute lymphoblastic leukemia
AML	acute myeloid leukemia
AMP	adenosine 5′-monophosphate
AMPK	AMP-activated protein kinase
ALS	amyotrophic lateral sclerosis
ATIC	AICAR formyltransferase/IMP cyclohydrolase
*Ascl1*	achaete-scute family bHLH transcription factor 1
BDNF	TrkB brain-derived neurotrophic factor
CD26	trimeric complexes dipeptidyl peptidase IV
CLL	chronic lymphoid leukemia
CN-I	cytosolic 5′-nucleotidase-I
CN-II	cytosolic 5′-nucleotidase II
CNS	central nervous system
*Darpp-32*	the gene encoding dopamine- and cAMP-regulated phosphoprotein 32
dCF	deoxycoformycin
ENT2	adenosine equilibrative transporters NBTI-insensitive
ES	embryonic stem cells
*Foxp1*	forkhead box P1
GMP	guanosine 5′-monophosphate
*HPRT*	the gene coding for HPRT
HPRT	hypoxanthine guanine phosphoribosyl transferase
HSPs	hereditary spastic paraplegias
IMP	inosine 5′-monophosphate
IMPDH	inosine-5′-monophosphate dehydrogenase
Ipaf	ice protease-activating factor
LND	Lesch–Nyhan disease
MAPK	mitogen activated protein kinases
6-MP	6-mercaptopurine
*NT5C2*	the gene coding for cN-II
NTPDases	5′-triphosphate diphosphohydrolases
NTPase	nucleoside 5-triphosphatase
PPAT	5-phosphoribosyl-1-pyrophosphate amidotransferase
PRPP	5-phosphoribosyl-1-pyrophosphate
P2XR	purinergic receptor subtype 2X
P2YR	purinergic receptor subtype 2Y
SAH	S-adenosylhomocysteine
SAHH	SAH hydrolase
SCID	severe combined immunodeficiency
SNPs	single nucleotide polymorphism
SP45	autosomic recessive spastic paraplegia 45
WNT4	wingless-type MMTV integration site family, member 4
XDH	xanthine dehydrogenase
XOD	xanthine oxidase

## References

[1] Pedley A.M., Benkovic S.J. (2017). A New View into the Regulation of Purine Metabolism: The Purinosome. Trends Biochem. Sci..

[2] Ipata P.L., Balestri F., Camici M., Tozzi M.G. (2011). Molecular mechanisms of nucleoside recycling in the brain. Int. J. Biochem. Cell Biol..

[3] Fridman A., Saha A., Chan A., Casteel D.E., Pilz R.B., Boss G.R. (2013). Cell cycle regulation of purine synthesis by phosphoribosyl pyrophosphate and inorganic phosphate. Biochem. J..

[4] An S., Kumar R., Sheets E.D., Benkovic S.J. (2008). Reversible compartmentalization of de novo purine biosynthetic complexes in living cells. Science.

[5] Zhou G., Smith J.L., Zalkin H. (1994). Binding of purine nucleotides to two regulatory sites results in synergistic feedback inhibition of glutamine 5-phosphoribosylpyrophosphate amidotransferase. J. Biol. Chem..

[6] Boer P., Sperling O. (1995). Role of cellular ribose-5-phosphate content in the regulation of 5-phosphoribosyl-1-pyrophosphate and de novo purine synthesis in a human hepatoma cell line. Metabolism.

[7] Camici M., Allegrini S., Tozzi M.G. (2018). Interplay between adenylate metabolizing enzymes and AMP-activated protein kinase. FEBS J..

[8] Tozzi M.G., Pesi R., Allegrini S. (2013). On the physiological role of cytosolic 5′-nucleotidase II (cN-II): Pathological and therapeutical implications. Curr. Med. Chem..

[9] Ipata P.L., Tozzi M.G. (2006). Recent advances in structure and function of cytosolic IMP-GMP specific 5′-nucleotidase II (cN-II). Purinergic Signal.

[10] Balestri F., Barsotti C., Lutzemberger L., Camici M., Ipata P.L. (2007). Key role of uridine kinase and uridine phosphorylase in the homeostatic regulation of purine and pyrimidine salvage in brain. Neurochem. Int..

[11] Pesi R., Petrotto E., Colombaioni L., Allegrini S., Garcia-Gil M., Camici M., Jordheim L.P., Tozzi M.G. (2018). Cytosolic 5′-Nucleotidase II Silencing in a Human Lung Carcinoma Cell Line Opposes Cancer Phenotype with a Concomitant Increase in p53 Phosphorylation. Int. J. Mol. Sci..

[12] Bricard G., Cadassou O., Cassagnes L.E., Cros-Perrial E., Payen-Gay L., Puy J.Y., Lefebvre-Tournier I., Tozzi M.G., Dumontet C., Jordheim L.P. (2017). The cytosolic 5′-nucleotidase cN-II lowers the adaptability to glucose deprivation in human breast cancer cells. Oncotarget.

[13] Cividini F., Cros-Perrial E., Pesi R., Machon C., Allegrini S., Camici M., Dumontet C., Jordheim L.P., Tozzi M.G. (2015). Cell proliferation and drug sensitivity of human glioblastoma cells are altered by the stable modulation of cytosolic 5′-nucleotidase II. Int. J. Biochem. Cell Biol..

[14] Cividini F., Filoni D.N., Pesi R., Allegrini S., Camici M., Tozzi M.G. (2015). IMP-GMP specific cytosolic 5′-nucleotidase regulates nucleotide pool and prodrug metabolism. Biochim. Biophys. Acta.

[15] Rampazzo C., Tozzi M.G., Dumontet C., Jordheim L.P. (2016). The druggability of intracellular nucleotide-degrading enzymes. Cancer Chemother. Pharmacol..

[16] Ipata P.L., Camici M., Micheli V., Tozz M.G. (2011). Metabolic network of nucleosides in the brain. Curr. Top. Med. Chem..

[17] Johansson M., Karlsson A. (1996). Cloning and expression of human deoxyguanosine kinase cDNA. Proc. Natl. Acad. Sci. USA.

[18] Barsotti C., Tozzi M.G., Ipata P.L. (2002). Purine and pyrimidine salvage in whole rat brain. Utilization of ATP-derived ribose-1-phosphate and 5-phosphoribosyl-1-pyrophosphate generated in experiments with dialyzed cell-free extracts. J. Biol. Chem..

[19] Schulte G., Fredholm B.B. (2003). Signalling from adenosine receptors to mitogen-activated protein kinases. Cell Signal.

[20] Abbracchio M.P., Burnstock G., Verkhratsky A., Zimmermann H. (2009). Purinergic signalling in the nervous system: An overview. Trends Neurosci..

[21] Boison D. (2008). Adenosine as a neuromodulator in neurological diseases. Curr. Opin. Pharmacol..

[22] Illes P., Verkhratsky A. (2016). Purinergic neurone-glia signalling in cognitive-related pathologies. Neuropharmacology.

[23] Burnstock G. (2017). Purinergic Signaling in the Cardiovascular System. Circ. Res..

[24] Cunha R.A. (2016). How does adenosine control neuronal dysfunction and neurodegeneration?. J. Neurochem..

[25] Fumagalli M., Lecca D., Abbracchio M.P., Ceruti S. (2017). Pathophysiological Role of Purines and Pyrimidines in Neurodevelopment: Unveiling New Pharmacological Approaches to Congenital Brain Diseases. Front. Pharmacol..

[26] Yue N., Huang H., Zhu X., Han Q., Wang Y., Li B., Liu Q., Wu G., Zhang Y., Yu J. (2017). Activation of P2X7 receptor and NLRP3 inflammasome assembly in hippocampal glial cells mediates chronic stress-induced depressive-like behaviors. J Neuroinflamm..

[27] Bhattacharya A. (2018). Recent Advances in CNS P2X7 Physiology and Pharmacology: Focus on Neuropsychiatric Disorders. Front. Pharmacol..

[28] Oliveira-Giacomelli A., Naaldijk Y., Sarda-Arroyo L., Goncalves M.C.B., Correa-Velloso J., Pillat M.M., de Souza H.D.N., Ulrich H. (2018). Purinergic Receptors in Neurological Diseases With Motor Symptoms: Targets for Therapy. Front. Pharmacol..

[29] Camici M., Garcia-Gil M., Tozzi M.G. (2018). The Inside Story of Adenosine. Int. J. Mol. Sci..

[30] Waring W.S. (2002). Uric acid: An important antioxidant in acute ischaemic stroke. QJM.

[31] Kutzing M.K., Firestein B.L. (2008). Altered uric acid levels and disease states. J. Pharmacol. Exp. Ther..

[32] Pasalic D., Marinkovic N., Feher-Turkovic L. (2012). Uric acid as one of the important factors in multifactorial disorders--facts and controversies. Biochem. Med..

[33] Cividini F., Tozzi M.G., Galli A., Pesi R., Camici M., Dumontet C., Jordheim L.P., Allegrini S. (2015). Cytosolic 5′-nucleotidase II interacts with the leucin rich repeat of NLR family member Ipaf. PLoS ONE.

[34] Dursun U., Koroglu C., Kocasoy Orhan E., Ugur S.A., Tolun A. (2009). Autosomal recessive spastic paraplegia (SPG45) with mental retardation maps to 10q24.3-q25.1. Neurogenetics.

[35] Novarino G., Fenstermaker A.G., Zaki M.S., Hofree M., Silhavy J.L., Heiberg A.D., Abdellateef M., Rosti B., Scott E., Mansour L. (2014). Exome sequencing links corticospinal motor neuron disease to common neurodegenerative disorders. Science.

[36] Elsaid M.F., Ibrahim K., Chalhoub N., Elsotouhy A., El Mudehki N., Abdel Aleem A. (2017). NT5C2 novel splicing variant expands the phenotypic spectrum of Spastic Paraplegia (SPG45): Case report of a new member of thin corpus callosum SPG-Subgroup. BMC Med. Genet..

[37] Straussberg R., Onoufriadis A., Konen O., Zouabi Y., Cohen L., Lee J.Y.W., Hsu C.K., Simpson M.A., McGrath J.A. (2017). Novel homozygous missense mutation in NT5C2 underlying hereditary spastic paraplegia SPG45. Am. J. Med. Genet. A.

[38] Darvish H., Azcona L.J., Tafakhori A., Ahmadi M., Ahmadifard A., Paisan-Ruiz C. (2017). Whole genome sequencing identifies a novel homozygous exon deletion in the NT5C2 gene in a family with intellectual disability and spastic paraplegia. NPJ Genom. Med..

[39] Pesi R., Micheli V., Jacomelli G., Peruzzi L., Camici M., Garcia-Gil M., Allegrini S., Tozzi M.G. (2000). Cytosolic 5′-nucleotidase hyperactivity in erythrocytes of Lesch-Nyhan syndrome patients. Neuroreport.

[40] Jordheim L.P., Chaloin L. (2013). Therapeutic perspectives for cN-II in cancer. Curr. Med. Chem..

[41] Mitra A.K., Kirstein M.N., Khatri A., Skubitz K.M., Dudek A.Z., Greeno E.W., Kratzke R.A., Lamba J.K. (2012). Pathway-based pharmacogenomics of gemcitabine pharmacokinetics in patients with solid tumors. Pharmacogenomics.

[42] Jordheim L.P., Puy J.Y., Cros-Perrial E., Peyrottes S., Lefebvre I., Perigaud C., Dumontet C. (2015). Determination of the enzymatic activity of cytosolic 5′-nucleotidase cN-II in cancer cells: Development of a simple analytical method and related cell line models. Anal. Bioanal. Chem..

[43] Tzoneva G., Dieck C.L., Oshima K., Ambesi-Impiombato A., Sanchez-Martin M., Madubata C.J., Khiabanian H., Yu J., Waanders E., Iacobucci I. (2018). Clonal evolution mechanisms in NT5C2 mutant-relapsed acute lymphoblastic leukaemia. Nature.

[44] Hnizda A., Fabry M., Moriyama T., Pachl P., Kugler M., Brinsa V., Ascher D.B., Carroll W.L., Novak P., Zaliova M. (2018). Relapsed acute lymphoblastic leukemia-specific mutations in NT5C2 cluster into hotspots driving intersubunit stimulation. Leukemia.

[45] Dieck C.L., Tzoneva G., Forouhar F., Carpenter Z., Ambesi-Impiombato A., Sanchez-Martin M., Kirschner-Schwabe R., Lew S., Seetharaman J., Tong L. (2018). Structure and Mechanisms of NT5C2 Mutations Driving Thiopurine Resistance in Relapsed Lymphoblastic Leukemia. Cancer Cell.

[46] Wallden K., Nordlund P. (2011). Structural basis for the allosteric regulation and substrate recognition of human cytosolic 5′-nucleotidase II. J. Mol. Biol..

[47] Studer F.E., Fedele D.E., Marowsky A., Schwerdel C., Wernli K., Vogt K., Fritschy J.M., Boison D. (2006). Shift of adenosine kinase expression from neurons to astrocytes during postnatal development suggests dual functionality of the enzyme. Neuroscience.

[48] Cui X.A., Singh B., Park J., Gupta R.S. (2009). Subcellular localization of adenosine kinase in mammalian cells: The long isoform of AdK is localized in the nucleus. Biochem. Biophys. Res. Commun..

[49] Shen H.Y., Lusardi T.A., Williams-Karnesky R.L., Lan J.Q., Poulsen D.J., Boison D. (2011). Adenosine kinase determines the degree of brain injury after ischemic stroke in mice. J. Cereb. Blood Flow Metab..

[50] Kiese K., Jablonski J., Boison D., Kobow K. (2016). Dynamic Regulation of the Adenosine Kinase Gene during Early Postnatal Brain Development and Maturation. Front. Mol. Neurosci..

[51] McNally T., Helfrich R.J., Cowart M., Dorwin S.A., Meuth J.L., Idler K.B., Klute K.A., Simmer R.L., Kowaluk E.A., Halbert D.N. (1997). Cloning and expression of the adenosine kinase gene from rat and human tissues. Biochem. Biophys. Res. Commun..

[52] Najmabadi H., Motazacker M.M., Garshasbi M., Kahrizi K., Tzschach A., Chen W., Behjati F., Hadavi V., Nieh S.E., Abedini S.S. (2007). Homozygosity mapping in consanguineous families reveals extreme heterogeneity of non-syndromic autosomal recessive mental retardation and identifies 8 novel gene loci. Hum. Genet..

[53] Bjursell M.K., Blom H.J., Cayuela J.A., Engvall M.L., Lesko N., Balasubramaniam S., Brandberg G., Halldin M., Falkenberg M., Jakobs C. (2011). Adenosine kinase deficiency disrupts the methionine cycle and causes hypermethioninemia, encephalopathy, and abnormal liver function. Am. J. Hum. Genet..

[54] Staufner C., Lindner M., Dionisi-Vici C., Freisinger P., Dobbelaere D., Douillard C., Makhseed N., Straub B.K., Kahrizi K., Ballhausen D. (2016). Adenosine kinase deficiency: Expanding the clinical spectrum and evaluating therapeutic options. J. Inherit. Metab. Dis..

[55] Shakiba M., Mahjoub F., Fazilaty H., Rezagholizadeh F., Shakiba A., Ziadlou M., Gahl W.A., Behnam B. (2016). Adenosine kinase deficiency with neurodevelopemental delay and recurrent hepatic dysfunction: A case report. Adv. Rare Dis..

[56] Boison D. (2016). Adenosinergic signaling in epilepsy. Neuropharmacology.

[57] de Groot M., Iyer A., Zurolo E., Anink J., Heimans J.J., Boison D., Reijneveld J.C., Aronica E. (2012). Overexpression of ADK in human astrocytic tumors and peritumoral tissue is related to tumor-associated epilepsy. Epilepsia.

[58] Huang J., He Y., Chen M., Du J., Li G., Li S., Liu W., Long X. (2015). Adenosine deaminase and adenosine kinase expression in human glioma and their correlation with gliomaassociated epilepsy. Mol. Med. Rep..

[59] Luan G., Gao Q., Guan Y., Zhai F., Zhou J., Liu C., Chen Y., Yao K., Qi X., Li T. (2013). Upregulation of adenosine kinase in Rasmussen encephalitis. J. Neuropathol. Exp. Neurol..

[60] Luan G., Wang X., Gao Q., Guan Y., Wang J., Deng J., Zhai F., Chen Y., Li T. (2017). Upregulation of Neuronal Adenosine A1 Receptor in Human Rasmussen Encephalitis. J. Neuropathol. Exp. Neurol..

[61] Luan G., Gao Q., Zhai F., Zhou J., Liu C., Chen Y., Li T. (2015). Adenosine kinase expression in cortical dysplasia with balloon cells: Analysis of developmental lineage of cell types. J. Neuropathol. Exp. Neurol..

[62] Sisodiya S.M., Fauser S., Cross J.H., Thom M. (2009). Focal cortical dysplasia type II: Biological features and clinical perspectives. Lancet Neurol..

[63] Boison D., Aronica E. (2015). Comorbidities in Neurology: Is adenosine the common link?. Neuropharmacology.

[64] Li T., Ren G., Lusardi T., Wilz A., Lan J.Q., Iwasato T., Itohara S., Simon R.P., Boison D. (2008). Adenosine kinase is a target for the prediction and prevention of epileptogenesis in mice. J. Clin. Investig..

[65] Singer P., McGarrity S., Shen H.Y., Boison D., Yee B.K. (2012). Working memory and the homeostatic control of brain adenosine by adenosine kinase. Neuroscience.

[66] Sandau U.S., Colino-Oliveira M., Jones A., Saleumvong B., Coffman S.Q., Liu L., Miranda-Lourenco C., Palminha C., Batalha V.L., Xu Y. (2016). Adenosine Kinase Deficiency in the Brain Results in Maladaptive Synaptic Plasticity. J. Neurosci..

[67] Williams-Karnesky R.L., Sandau U.S., Lusardi T.A., Lytle N.K., Farrell J.M., Pritchard E.M., Kaplan D.L., Boison D. (2013). Epigenetic changes induced by adenosine augmentation therapy prevent epileptogenesis. J. Clin. Investig..

[68] James S.J., Melnyk S., Pogribna M., Pogribny I.P., Caudill M.A. (2002). Elevation in S-adenosylhomocysteine and DNA hypomethylation: Potential epigenetic mechanism for homocysteine-related pathology. J. Nutr..

[69] Diamond M.L., Ritter A.C., Jackson E.K., Conley Y.P., Kochanek P.M., Boison D., Wagner A.K. (2015). Genetic variation in the adenosine regulatory cycle is associated with posttraumatic epilepsy development. Epilepsia.

[70] Poppe D., Doerr J., Schneider M., Wilkens R., Steinbeck J.A., Ladewig J., Tam A., Paschon D.E., Gregory P.D., Reik A. (2018). Genome Editing in Neuroepithelial Stem Cells to Generate Human Neurons with High Adenosine-Releasing Capacity. Stem Cells Transl. Med..

[71] Sai K., Yang D., Yamamoto H., Fujikawa H., Yamamoto S., Nagata T., Saito M., Yamamura T., Nishizaki T. (2006). A(1) adenosine receptor signal and AMPK involving caspase-9/-3 activation are responsible for adenosine-induced RCR-1 astrocytoma cell death. Neurotoxicology.

[72] Ohkubo S., Nagata K., Nakahata N. (2007). Adenosine uptake-dependent C6 cell growth inhibition. Eur. J. Pharmacol..

[73] Rocha R., Torres A., Ojeda K., Uribe D., Rocha D., Erices J., Niechi I., Ehrenfeld P., San Martin R., Quezada C. (2018). The Adenosine A(3) Receptor Regulates Differentiation of Glioblastoma Stem-Like Cells to Endothelial Cells under Hypoxia. Int. J. Mol. Sci..

[74] Torres A., Vargas Y., Uribe D., Jaramillo C., Gleisner A., Salazar-Onfray F., Lopez M.N., Melo R., Oyarzun C., San Martin R. (2016). Adenosine A3 receptor elicits chemoresistance mediated by multiple resistance-associated protein-1 in human glioblastoma stem-like cells. Oncotarget.

[75] Siebel A.M., Piato A.L., Schaefer I.C., Nery L.R., Bogo M.R., Bonan C.D. (2013). Antiepileptic drugs prevent changes in adenosine deamination during acute seizure episodes in adult zebrafish. Pharmacol. Biochem. Behav..

[76] Wahlman C., Doyle T.M., Little J.W., Luongo L., Janes K., Chen Z., Esposito E., Tosh D.K., Cuzzocrea S., Jacobson K.A. (2018). Chemotherapy-induced pain is promoted by enhanced spinal adenosine kinase levels through astrocyte-dependent mechanisms. Pain.

[77] Whitmore K.V., Gaspar H.B. (2016). Adenosine Deaminase Deficiency—More Than Just an Immunodeficiency. Front. Immunol..

[78] Flinn A.M., Gennery A.R. (2018). Adenosine deaminase deficiency: A review. Orphanet J. Rare Dis..

[79] Rogers M.H., Lwin R., Fairbanks L., Gerritsen B., Gaspar H.B. (2001). Cognitive and behavioral abnormalities in adenosine deaminase deficient severe combined immunodeficiency. J. Pediatr..

[80] Nofech-Mozes Y., Blaser S.I., Kobayashi J., Grunebaum E., Roifman C.M. (2007). Neurologic abnormalities in patients with adenosine deaminase deficiency. Pediatr Neurol.

[81] Titman P., Pink E., Skucek E., O’Hanlon K., Cole T.J., Gaspar J., Xu-Bayford J., Jones A., Thrasher A.J., Davies E.G. (2008). Cognitive and behavioral abnormalities in children after hematopoietic stem cell transplantation for severe congenital immunodeficiencies. Blood.

[82] Stubbs G., Litt M., Lis E., Jackson R., Voth W., Lindberg A., Litt R. (1982). Adenosine deaminase activity decreased in autism. J. Am. Acad. Child Psychiatry.

[83] Bottini N., De Luca D., Saccucci P., Fiumara A., Elia M., Porfirio M.C., Lucarelli P., Curatolo P. (2001). Autism: Evidence of association with adenosine deaminase genetic polymorphism. Neurogenetics.

[84] Saccucci P., Arpino C., Rizzo R., Gagliano A., Volzone A., Lalli C., Galasso C., Curatolo P. (2006). Association of adenosine deaminase polymorphism with mild mental retardation. J. Child Neurol..

[85] Honig M., Albert M.H., Schulz A., Sparber-Sauer M., Schutz C., Belohradsky B., Gungor T., Rojewski M.T., Bode H., Pannicke U. (2007). Patients with adenosine deaminase deficiency surviving after hematopoietic stem cell transplantation are at high risk of CNS complications. Blood.

[86] Booth C., Gaspar H.B. (2009). Pegademase bovine (PEG-ADA) for the treatment of infants and children with severe combined immunodeficiency (SCID). Biologics.

[87] Cicalese M.P., Ferrua F., Castagnaro L., Pajno R., Barzaghi F., Giannelli S., Dionisio F., Brigida I., Bonopane M., Casiraghi M. (2016). Update on the safety and efficacy of retroviral gene therapy for immunodeficiency due to adenosine deaminase deficiency. Blood.

[88] Sauer A.V., Hernandez R.J., Fumagalli F., Bianchi V., Poliani P.L., Dallatomasina C., Riboni E., Politi L.S., Tabucchi A., Carlucci F. (2017). Alterations in the brain adenosine metabolism cause behavioral and neurological impairment in ADA-deficient mice and patients. Sci. Rep..

[89] Ganguly P., Brenhouse H.C. (2015). Broken or maladaptive? Altered trajectories in neuroinflammation and behavior after early life adversity. Dev. Cognit. Neurosci..

[90] Blackburn M.R., Datta S.K., Kellems R.E. (1998). Adenosine deaminase-deficient mice generated using a two-stage genetic engineering strategy exhibit a combined immunodeficiency. J. Biol. Chem..

[91] Ledent C., Vaugeois J.M., Schiffmann S.N., Pedrazzini T., El Yacoubi M., Vanderhaeghen J.J., Costentin J., Heath J.K., Vassart G., Parmentier M. (1997). Aggressiveness, hypoalgesia and high blood pressure in mice lacking the adenosine A2a receptor. Nature.

[92] Ciruela F., Albergaria C., Soriano A., Cuffi L., Carbonell L., Sanchez S., Gandia J., Fernandez-Duenas V. (2010). Adenosine receptors interacting proteins (ARIPs): Behind the biology of adenosine signaling. Biochim. Biophys. Acta.

[93] Gracia E., Perez-Capote K., Moreno E., Barkesova J., Mallol J., Lluis C., Franco R., Cortes A., Casado V., Canela E.I. (2011). A2A adenosine receptor ligand binding and signalling is allosterically modulated by adenosine deaminase. Biochem. J..

[94] Gracia E., Cortes A., Meana J.J., Garcia-Sevilla J., Herhsfield M.S., Canela E.I., Mallol J., Lluis C., Franco R., Casado V. (2008). Human adenosine deaminase as an allosteric modulator of human A(1) adenosine receptor: Abolishment of negative cooperativity for [H](R)-pia binding to the caudate nucleus. J. Neurochem..

[95] Antonioli L., Fornai M., Awwad O., Giustarini G., Pellegrini C., Tuccori M., Caputi V., Qesari M., Castagliuolo I., Brun P. (2014). Role of the A(2B) receptor-adenosine deaminase complex in colonic dysmotility associated with bowel inflammation in rats. Br. J. Pharmacol..

[96] Moreno E., Canet J., Gracia E., Lluis C., Mallol J., Canela E.I., Cortes A., Casado V. (2018). Molecular Evidence of Adenosine Deaminase Linking Adenosine A2A Receptor and CD26 Proteins. Front. Pharmacol..

[97] Havre P.A., Abe M., Urasaki Y., Ohnuma K., Morimoto C., Dang N.H. (2008). The role of CD26/dipeptidyl peptidase IV in cancer. Front. Biosci..

[98] Kulkarni J.S., Wakade A.R. (1996). Quantitative analysis of similarities and differences in neurotoxicities caused by adenosine and 2′-deoxyadenosine in sympathetic neurons. J. Neurochem..

[99] Wakade A.R., Guo X., Palmer K.C., Kulkarni J.S., Przywara D.A., Wakade T.D. (1996). 2′-deoxyadenosine induces apoptosis in rat chromaffin cells. J. Neurochem..

[100] Garcia-Gil M., Tozzi M.G., Allegrini S., Folcarelli S., Della Sala G., Voccoli V., Colombaioni L., Camici M. (2012). Novel metabolic aspects related to adenosine deaminase inhibition in a human astrocytoma cell line. Neurochem. Int..

[101] Garcia-Gil M., Tozzi M.G., Varani S., Della Verde L., Petrotto E., Balestri F., Colombaioni L., Camici M. (2015). The combination of adenosine deaminase inhibition and deoxyadenosine induces apoptosis in a human astrocytoma cell line. Neurochem. Int..

[102] Garcia-Gil M., Tozzi M.G., Balestri F., Colombaioni L., Camici M. (2016). Mitochondrial Damage and Apoptosis Induced by Adenosine Deaminase Inhibition and Deoxyadenosine in Human Neuroblastoma Cell Lines. J. Cell. Biochem..

[103] Ng S.K., Higashimori H., Tolman M., Yang Y. (2015). Suppression of adenosine 2a receptor (A2aR)-mediated adenosine signaling improves disease phenotypes in a mouse model of amyotrophic lateral sclerosis. Exp. Neurol..

[104] Agarwal R.P., Spector T., Parks R.E. (1977). Tight-Binding Inhibitors. 4. Inhibition of Adenosine Deaminases by Various Inhibitors. Biochem. Pharmacol..

[105] Dohner H., Ho A.D., Thaler J., Stryckmans P., Sonneveld P., de Witte T., Lechner K., Lauria F., Bodewadt-Radzun S., Suciu S. (1993). Pentostatin in prolymphocytic leukemia: Phase II trial of the European Organization for Research and Treatment of Cancer Leukemia Cooperative Study Group. J. Natl. Cancer Inst..

[106] Willis C.R., Goodrich A., Park K., Waselenko J.K., Lucas M., Reese A., Diehl L.F., Grever M.R., Byrd J.C., Flinn I.W. (2006). A phase I/II study examining pentostatin, chlorambucil, and theophylline in patients with relapsed chronic lymphocytic leukemia and non-Hodgkin’s lymphoma. Ann. Hematol..

[107] Kay N.E., LaPlant B.R., Pettinger A.M., Call T.G., Leis J.F., Ding W., Parikh S.A., Conte M.J., Bowen D.A., Shanafelt T.D. (2018). Cumulative experience and long term follow-up of pentostatin-based chemoimmunotherapy trials for patients with chronic lymphocytic leukemia. Expert Rev. Hematol..

[108] Tedeschi A., Rossi D., Motta M., Quaresmini G., Rossi M., Coscia M., Anastasia A., Rossini F., Cortelezzi A., Nador G. (2015). A phase II multi-center trial of pentostatin plus cyclophosphamide with ofatumumab in older previously untreated chronic lymphocytic leukemia patients. Haematologica.

[109] Hunt S.W., Hoffee P.A. (1982). Adenosine deaminase from deoxycoformycin-sensitive and -resistant rat hepatoma cells. Purification and characterization. J. Biol. Chem..

[110] Camici M., Turriani M., Tozzi M.G., Turchi G., Cos J., Alemany C., Miralles A., Noe V., Ciudad C.J. (1995). Purine enzyme profile in human colon-carcinoma cell lines and differential sensitivity to deoxycoformycin and 2′-deoxyadenosine in combination. Int. J. Cancer.

[111] Bemi V., Tazzni N., Banditelli S., Giorgelli F., Pesi R., Turchi G., Mattana A., Sgarrella F., Tozzi M.G., Camici M. (1998). Deoxyadenosine metabolism in a human colon-carcinoma cell line (LoVo) in relation to its cytotoxic effect in combination with deoxycoformycin. Int. J. Cancer.

[112] Giannecchini M., D’Innocenzo B., Pesi R., Sgarrella F., Iorio M., Collecchi P., Tozzi M.G., Camici M. (2003). 2′-Deoxyadenosine causes apoptotic cell death in a human colon carcinoma cell line. J. Biochem. Mol. Toxicol..

[113] Kutryb-Zajac B., Koszalka P., Mierzejewska P., Bulinska A., Zabielska M.A., Brodzik K., Skrzypkowska A., Zelazek L., Pelikant-Malecka I., Slominska E.M. (2018). Adenosine deaminase inhibition suppresses progression of 4T1 murine breast cancer by adenosine receptor-dependent mechanisms. J. Cell. Mol. Med..

[114] Micheli V., Camici M., Tozzi M.G., Ipata P.L., Sestini S., Bertelli M., Pompucci G. (2011). Neurological disorders of purine and pyrimidine metabolism. Curr. Top. Med. Chem..

[115] Deutsch S.I., Long K.D., Rosse R.B., Mastropaolo J., Eller J. (2005). Hypothesized deficiency of guanine-based purines may contribute to abnormalities of neurodevelopment, neuromodulation, and neurotransmission in Lesch-Nyhan syndrome. Clin. Neuropharmacol..

[116] Ceballos-Picot I., Mockel L., Potier M.C., Dauphinot L., Shirley T.L., Torero-Ibad R., Fuchs J., Jinnah H.A. (2009). Hypoxanthine-guanine phosphoribosyl transferase regulates early developmental programming of dopamine neurons: Implications for Lesch-Nyhan disease pathogenesis. Hum. Mol. Genet..

[117] Ernst M., Zametkin A.J., Matochik J.A., Pascualvaca D., Jons P.H., Hardy K., Hankerson J.G., Doudet D.J., Cohen R.M. (1996). Presynaptic dopaminergic deficits in Lesch-Nyhan disease. N. Engl. J. Med..

[118] Wong D.F., Harris J.C., Naidu S., Yokoi F., Marenco S., Dannals R.F., Ravert H.T., Yaster M., Evans A., Rousset O. (1996). Dopamine transporters are markedly reduced in Lesch-Nyhan disease in vivo. Proc. Natl. Acad. Sci. USA.

[119] Schretlen D.J., Varvaris M., Vannorsdall T.D., Gordon B., Harris J.C., Jinnah H.A. (2015). Brain white matter volume abnormalities in Lesch-Nyhan disease and its variants. Neurology.

[120] Torres R.J., Prior C., Garcia M.G., Puig J.G. (2016). A review of the implication of hypoxanthine excess in the physiopathology of Lesch-Nyhan disease. Nucleosides Nucleotides Nucleic Acids.

[121] Pelled D., Sperling O., Zoref-Shani E. (1999). Abnormal purine and pyrimidine nucleotide content in primary astroglia cultures from hypoxanthine-guanine phosphoribosyltransferase-deficient transgenic mice. J. Neurochem..

[122] Zoref-Shani E., Bromberg Y., Brosh S., Sidi Y., Sperling O. (1993). Characterization of the alterations in purine nucleotide metabolism in hypoxanthine-guanine phosphoribosyltransferase-deficient rat neuroma cell line. J. Neurochem..

[123] Shirley T.L., Lewers J.C., Egami K., Majumdar A., Kelly M., Ceballos-Picot I., Seidman M.M., Jinnah H.A. (2007). A human neuronal tissue culture model for Lesch-Nyhan disease. J. Neurochem..

[124] Fu R., Sutcliffe D., Zhao H., Huang X., Schretlen D.J., Benkovic S., Jinnah H.A. (2015). Clinical severity in Lesch-Nyhan disease: The role of residual enzyme and compensatory pathways. Mol. Genet. Metab..

[125] Ma M.H., Stacey N.C., Connolly G.P. (2001). Hypoxanthine impairs morphogenesis and enhances proliferation of a neuroblastoma model of Lesch Nyhan syndrome. J. Neurosci. Res..

[126] Asano T., Spector S. (1979). Identification of Inosine and Hypoxanthine as Endogenous Ligands for the Brain Benzodiazepine-Binding Sites. Proc. Natl. Acad. Sci. USA.

[127] Goldstein M., Anderson L.T., Reuben R., Dancis J. (1985). Self-mutilation in Lesch-Nyhan disease is caused by dopaminergic denervation. Lancet.

[128] Torres R.J., Puig J.G. (2015). Hypoxanthine deregulates genes involved in early neuronal development. Implications in Lesch-Nyhan disease pathogenesis. J. Inherit. Metab. Dis..

[129] Garcia M.G., Puig J.G., Torres R.J. (2012). Adenosine, dopamine and serotonin receptors imbalance in lymphocytes of Lesch-Nyhan patients. J. Inherit. Metab. Dis..

[130] Frizzo M.E., Antunes Soares F.A., Dall’Onder L.P., Lara D.R., Swanson R.A., Souza D.O. (2003). Extracellular conversion of guanine-based purines to guanosine specifically enhances astrocyte glutamate uptake. Brain Res..

[131] Brosh S., Sperling O., Dantziger E., Sidi Y. (1992). Metabolism of guanine and guanine nucleotides in primary rat neuronal cultures. J. Neurochem..

[132] Torres R.J., Deantonio I., Prior C., Puig J.G. (2004). Adenosine transport in peripheral blood lymphocytes from Lesch-Nyhan patients. Biochem. J..

[133] Prior C., Torres R.J., Puig J.G. (2006). Hypoxanthine effect on equilibrative and concentrative adenosine transport in human lymphocytes: Implications in the phatogenesis of Lesch-Nyhan syndrome. Nucleosides Nucleotides Nucleic Acids.

[134] Park T.S., Gidday J.M. (1990). Effect of dipyridamole on cerebral extracellular adenosine level in vivo. J. Cereb. Blood Flow Metab..

[135] Phillis J.W., O’Regan M.H., Walter G.A. (1989). Effects of two nucleoside transport inhibitors, dipyridamole and soluflazine, on purine release from the rat cerebral cortex. Brain Res..

[136] Newby A.C. (1986). How does dipyridamole elevate extracellular adenosine concentration? Predictions from a three-compartment model of adenosine formation and inactivation. Biochem. J..

[137] Biasibetti H., Pierozan P., Rodrigues A.F., Manfredini V., Wyse A.T.S. (2017). Hypoxanthine Intrastriatal Administration Alters Neuroinflammatory Profile and Redox Status in Striatum of Infant and Young Adult Rats. Mol. Neurobiol..

[138] Biasibetti-Brendler H., Schmitz F., Pierozan P., Zanotto B.S., Prezzi C.A., de Andrade R.B., Wannmacher C.M.D., Wyse A.T.S. (2018). Hypoxanthine Induces Neuroenergetic Impairment and Cell Death in Striatum of Young Adult Wistar Rats. Mol. Neurobiol..

[139] Sidi Y., Mitchell B.S. (1985). Z-nucleotide accumulation in erythrocytes from Lesch-Nyhan patients. J. Clin. Investig..

[140] Marie S., Heron B., Bitoun P., Timmerman T., Van Den Berghe G., Vincent M.F. (2004). AICA-ribosiduria: A novel, neurologically devastating inborn error of purine biosynthesis caused by mutation of ATIC. Am. J. Hum. Genet..

[141] Corton J.M., Gillespie J.G., Hawley S.A., Hardie D.G. (1995). 5-aminoimidazole-4-carboxamide ribonucleoside. A specific method for activating AMP-activated protein kinase in intact cells?. Eur. J. Biochem..

[142] Garcia-Gil M., Pesi R., Perna S., Allegrini S., Giannecchini M., Camici M., Tozzi M.G. (2003). 5′-aminoimidazole-4-carboxamide riboside induces apoptosis in human neuroblastoma cells. Neuroscience.

[143] Lopez J.M. (2008). Is ZMP the toxic metabolite in Lesch-Nyhan disease?. Med. Hypotheses.

[144] Fukuda T., Ishii K., Nanmoku T., Isobe K., Kawakami Y., Takekoshi K. (2007). 5-Aminoimidazole-4-carboxamide-1-beta-4-ribofuranoside stimulates tyrosine hydroxylase activity and catecholamine secretion by activation of AMP-activated protein kinase in PC12 cells. J. Neuroendocrinol..

[145] Chan C.Y., Zhao H., Pugh R.J., Pedley A.M., French J., Jones S.A., Zhuang X., Jinnah H., Huang T.J., Benkovic S.J. (2015). Purinosome formation as a function of the cell cycle. Proc. Natl. Acad. Sci. USA.

[146] Pinto C.S., Jinnah H.A., Shirley T.L., Nyhan W.L., Seifert R. (2005). Altered membrane NTPase activity in Lesch-Nyhan disease fibroblasts: Comparison with HPRT knockout mice and HPRT-deficient cell lines. J. Neurochem..

[147] Lorenz V., Pinto C.S., Seifert R. (2007). Complex changes in ecto-nucleoside 5′-triphosphate diphosphohydrolase expression in hypoxanthine phosphoribosyl transferase deficiency. Neurosci. Lett..

[148] Erdorf M., von der Ohe J., Seifert R. (2011). Impaired P2X and P2Y receptor-mediated signaling in HPRT-deficient B103 neuroblastoma cells. Neurosci. Lett..

[149] Mastrangelo L., Kim J.E., Miyanohara A., Kang T.H., Friedmann T. (2012). Purinergic signaling in human pluripotent stem cells is regulated by the housekeeping gene encoding hypoxanthine guanine phosphoribosyltransferase. Proc. Natl. Acad. Sci. USA.

[150] Kyriakis J.M., Avruch J. (2001). Mammalian mitogen-activated protein kinase signal transduction pathways activated by stress and inflammation. Physiol. Rev..

[151] Kang T.H., Park Y., Bader J.S., Friedmann T. (2013). The housekeeping gene hypoxanthine guanine phosphoribosyltransferase (HPRT) regulates multiple developmental and metabolic pathways of murine embryonic stem cell neuronal differentiation. PLoS ONE.

[152] Ellis P., Fagan B.M., Magness S.T., Hutton S., Taranova O., Hayashi S., McMahon A., Rao M., Pevny L. (2004). SOX2, a persistent marker for multipotential neural stem cells derived from embryonic stem cells, the embryo or the adult. Dev. Neurosci..

[153] Guibinga G.H., Barron N., Pandori W. (2014). Striatal neurodevelopment is dysregulated in purine metabolism deficiency and impacts DARPP-32, BDNF/TrkB expression and signaling: New insights on the molecular and cellular basis of Lesch-Nyhan Syndrome. PLoS ONE.

[154] Guibinga G.H., Hrustanovic G., Bouic K., Jinnah H.A., Friedmann T. (2012). MicroRNA-mediated dysregulation of neural developmental genes in HPRT deficiency: Clues for Lesch-Nyhan disease?. Hum. Mol. Genet..

[155] Kang T.H., Friedmann T. (2015). Alzheimer’s disease shares gene expression aberrations with purinergic dysregulation of HPRT deficiency (Lesch-Nyhan disease). Neurosci. Lett..

[156] Camici M., Tozzi M.G., Allegrini S., Del Corso A., Sanfilippo O., Daidone M.G., De Marco C., Ipata P.L. (1990). Purine salvage enzyme activities in normal and neoplastic human tissues. Cancer Biochem. Biophys..

[157] Sanfilippo O., Camici M., Tozzi M.G., Turriani M., Faranda A., Ipata P.L., Silvestrini R. (1994). Relationship between the levels of purine salvage pathway enzymes and clinical/biological aggressiveness of human colon carcinoma. Cancer Biochem. Biophys..

[158] Muller A., Homey B., Soto H., Ge N., Catron D., Buchanan M.E., McClanahan T., Murphy E., Yuan W., Wagner S.N. (2001). Involvement of chemokine receptors in breast cancer metastasis. Nature.

[159] Townsend M.H., Felsted A.M., Ence Z.E., Piccolo S.R., Robison R.A., O’Neill K.L. (2017). Elevated Expression of Hypoxanthine Guanine Phosphoribosyltransferase within Malignant Tissue. Cancer Clin. Oncol..

[160] Townsend M.H., Anderson M.D., Weagel E.G., Velazquez E.J., Weber K.S., Robison R.A., O’Neill K.L. (2017). Non-small-cell lung cancer cell lines A549 and NCI-H460 express hypoxanthine guanine phosphoribosyltransferase on the plasma membrane. OncoTargets Ther..

[161] Townsend M.H., Robison R.A., O’Neill K.L. (2018). A review of HPRT and its emerging role in cancer. Med. Oncol..

[162] Hille R., Nishino T. (1995). Flavoprotein structure and mechanism. 4. Xanthine oxidase and xanthine dehydrogenase. FASEB J..

[163] Nishino T., Okamoto K., Eger B.T., Pai E.F., Nishino T. (2008). Mammalian xanthine oxidoreductase—Mechanism of transition from xanthine dehydrogenase to xanthine oxidase. FEBS J..

[164] Battelli M.G., Bortolotti M., Polito L., Bolognesi A. (2018). The role of xanthine oxidoreductase and uric acid in metabolic syndrome. Biochim. Biophys. Acta.

[165] So A., Thorens B. (2010). Uric acid transport and disease. J. Clin. Investig..

[166] Vorbach C., Scriven A., Capecchi M.R. (2002). The housekeeping gene xanthine oxidoreductase is necessary for milk fat droplet enveloping and secretion: Gene sharing in the lactating mammary gland. Genes Dev..

[167] Crane J.K., Naeher T.M., Broome J.E., Boedeker E.C. (2013). Role of host xanthine oxidase in infection due to enteropathogenic and Shiga-toxigenic Escherichia coli. Infect. Immun..

[168] Simeunovic Ostojic M., Maas J. (2018). Anorexia nervosa and uric acid beyond gout: An idea worth researching. Int. J. Eat Disord..

[169] Davies K.J., Sevanian A., Muakkassah-Kelly S.F., Hochstein P. (1986). Uric acid-iron ion complexes. A new aspect of the antioxidant functions of uric acid. Biochem. J..

[170] Alvarez-Lario B., Macarron-Vicente J. (2011). Is there anything good in uric acid?. QJM.

[171] Jackson E.K., Boison D., Schwarzschild M.A., Kochanek P.M. (2016). Purines: Forgotten mediators in traumatic brain injury. J. Neurochem..

[172] Mount D.B., Kwon C.Y., Zandi-Nejad K. (2006). Renal urate transport. Rheum. Dis. Clin..

[173] Hisatome I., Tsuboi M., Shigemasa C. (1996). Renal hypouricemia. Nihon Rinsho.

[174] Sharaf El Din U.A.A., Salem M.M., Abdulazim D.O. (2017). Uric acid in the pathogenesis of metabolic, renal, and cardiovascular diseases: A review. J. Adv. Res..

[175] Facchini F., Chen Y.D.I., Hollenbeck C.B., Reaven G.M. (1991). Relationship between Resistance to Insulin-Mediated Glucose-Uptake, Urinary Uric-Acid Clearance, and Plasma Uric-Acid Concentration. J. Am. Med. Assoc..

[176] Kang D.H., Chen W. (2011). Uric acid and chronic kidney disease: New understanding of an old problem. Semin. Nephrol..

[177] Perheentupa J., Raivio K. (1967). Fructose-induced hyperuricaemia. Lancet.

[178] Bjorge T., Lukanova A., Jonsson H., Tretli S., Ulmer H., Manjer J., Stocks T., Selmer R., Nagel G., Almquist M. (2010). Metabolic syndrome and breast cancer in the me-can (metabolic syndrome and cancer) project. Cancer Epidemiol. Biomark. Prev..

[179] Rose D.P., Haffner S.M., Baillargeon J. (2007). Adiposity, the metabolic syndrome, and breast cancer in African-American and white American women. Endocr. Rev..

[180] Giovannucci E. (2007). Metabolic syndrome, hyperinsulinemia, and colon cancer: A review. Am. J. Clin. Nutr..

[181] Fini M.A., Elias A., Johnson R.J., Wright R.M. (2012). Contribution of uric acid to cancer risk, recurrence, and mortality. Clin. Transl. Med..

[182] Mazzali M., Kanbay M., Segal M.S., Shafiu M., Jalal D., Feig D.I., Johnson R.J. (2010). Uric acid and hypertension: Cause or effect?. Curr. Rheumatol. Rep..

[183] Sofaer J.A., Emery A.E. (1981). Genes for super-intelligence?. J. Med. Genet..

[184] Orowan E. (1955). The origin of man. Nature.

[185] Cervini C., Zampa A.M. (1982). Uric-Acid and Intelligence. Ann. Rheum. Dis..

[186] Inouye E., Park K.S., Asaka A. (1984). Blood uric acid level and IQ: A study in twin families. Acta Genet. Med. Gemellol..

[187] Scott G.S., Hooper D.C. (2001). The role of uric acid in protection against peroxynitrite-mediated pathology. Med. Hypotheses.

[188] Kori M., Aydin B., Unal S., Arga K.Y., Kazan D. (2016). Metabolic Biomarkers and Neurodegeneration: A Pathway Enrichment Analysis of Alzheimer’s Disease, Parkinson’s Disease, and Amyotrophic Lateral Sclerosis. OMICS.

[189] Sian J., Dexter D.T., Lees A.J., Daniel S., Agid Y., Javoy-Agid F., Jenner P., Marsden C.D. (1994). Alterations in glutathione levels in Parkinson’s disease and other neurodegenerative disorders affecting basal ganglia. Ann. Neurol..

[190] de Lau L.M., Koudstaal P.J., Hofman A., Breteler M.M. (2005). Serum uric acid levels and the risk of Parkinson disease. Ann. Neurol..

[191] Lolekha P., Wongwan P., Kulkantrakorn K. (2015). Association between serum uric acid and motor subtypes of Parkinson’s disease. J. Clin. Neurosci..

[192] Huang X., Ng S.Y., Chia N.S., Acharyya S., Setiawan F., Lu Z.H., Ng E., Tay K.Y., Au W.L., Tan E.K. (2018). Serum uric acid level and its association with motor subtypes and non-motor symptoms in early Parkinson’s disease: PALS study. Parkinsonism Relat. Disord..

[193] Wu D., Wang L., Teng W., Huang K., Shang X. (2014). Correlation of fatigue during the acute stage of stroke with serum uric acid and glucose levels, depression, and disability. Eur. Neurol..

[194] Naviaux R.K., Naviaux J.C., Li K., Bright A.T., Alaynick W.A., Wang L., Baxter A., Nathan N., Anderson W., Gordon E. (2016). Metabolic features of chronic fatigue syndrome. Proc. Natl. Acad. Sci. USA.

[195] Huang T.T., Hao D.L., Wu B.N., Mao L.L., Zhang J. (2017). Uric acid demonstrates neuroprotective effect on Parkinson’s disease mice through Nrf2-ARE signaling pathway. Biochem. Biophys. Res. Commun..

[196] Keizman D., Ish-Shalom M., Berliner S., Maimon N., Vered Y., Artamonov I., Tsehori J., Nefussy B., Drory V.E. (2009). Low uric acid levels in serum of patients with ALS: Further evidence for oxidative stress?. J. Neurol. Sci..

[197] Kato S., Kato M., Kusano T., Nishino T. (2016). New Strategy That Delays Progression of Amyotrophic Lateral Sclerosis in G1H-G93A Transgenic Mice: Oral Administration of Xanthine Oxidoreductase Inhibitors That Are Not Substrates for the Purine Salvage Pathway. J. Neuropathol. Exp. Neurol..

[198] Chamorro A., Obach V., Cervera A., Revilla M., Deulofeu R., Aponte J.H. (2002). Prognostic significance of uric acid serum concentration in patients with acute ischemic stroke. Stroke.

[199] Chamorro A., Amaro S., Castellanos M., Segura T., Arenillas J., Marti-Fabregas J., Gallego J., Krupinski J., Gomis M., Canovas D. (2014). Safety and efficacy of uric acid in patients with acute stroke (URICO-ICTUS): A randomised, double-blind phase 2b/3 trial. Lancet Neurol..

[200] Schwarzschild M.A., Macklin E.A., Ascherio A., Parkinson Study Group SURE-PD Investigators (2014). Urate and neuroprotection trials. Lancet Neurol..

[201] Dachir S., Shabashov D., Trembovler V., Alexandrovich A.G., Benowitz L.I., Shohami E. (2014). Inosine improves functional recovery after experimental traumatic brain injury. Brain Res..

[202] Kim D., Zai L., Liang P., Schaffling C., Ahlborn D., Benowitz L.I. (2013). Inosine enhances axon sprouting and motor recovery after spinal cord injury. PLoS ONE.

[203] Shen H., Chen G.J., Harvey B.K., Bickford P.C., Wang Y. (2005). Inosine reduces ischemic brain injury in rats. Stroke.

